# Fundamental and unique roles of PLAC1 in the regulation of rat and human trophoblast cell development

**DOI:** 10.1242/dev.205290

**Published:** 2026-05-12

**Authors:** Ayelen Moreno-Irusta, Jovana Urosevic, Khursheed Iqbal, Arun S. Seetharam, Jackson Nteeba, Regan L. Scott, Marija Kuna, Masanaga Muto, Keisuke Kozai, Andjelka Celic, Hiroaki Okae, Takahiro Arima, David K. Johnson, Geetu Tuteja, Michael J. Soares

**Affiliations:** ^1^ Institute for Reproductive and Developmental Sciences, Department of Pathology and Laboratory Medicine, University of Kansas Medical Center, Kansas City, KS 66160, USA; ^2^ Reproductive Biology Program, University of Novi Sad, Novi Sad, Serbia; ^3^ Department of Genetics, Development, and Cell Biology, Iowa State University, Ames, IA 50011, USA; ^4^ Department of Trophoblast Research, Institute of Molecular Embryology and Genetics, Kumamoto University, Kumamoto 860-0811, Japan; ^5^ Department of Informative Genetics, Environment and Genome Research Center, Tohoku University Graduate School of Medicine, and Department of Molecular Oncology, Institute of Development, Aging and Cancer, Tohoku University, Sendai 980-8575, Japan; ^6^ Computational Chemical Biology Core and the Molecular Graphics and Modeling Laboratory, University of Kansas, Lawrence, KS 66047, USA; ^7^ Department of Obstetrics and Gynecology, University of Kansas Medical Center, Kansas City, KS 66160, USA

**Keywords:** Placenta, Trophoblast cells, PLAC1, FURIN

## Abstract

Placenta enriched 1 (PLAC1) is a conserved X chromosome-linked gene expressed in the mammalian placenta. We investigated the biology of PLAC1 in the rat and human placenta. *Plac1* transcripts were expressed in the junctional zone of the rat placenta and in intrauterine invasive trophoblast cells. Genome-edited *Plac1* mutant animals exhibited placentomegaly. Enlarged placentas were characterized by an expanded junctional zone, an irregular junctional zone-labyrinth zone boundary, a deficiency of intrauterine invasive trophoblast cells, and a late-gestation-stage uterine-placental interface infiltrated with natural killer cells. PLAC1 facilitated rat trophoblast cell differentiation. In contrast, PLAC1 showed minimal contributions to the regulation of the human invasive/extravillous trophoblast cell lineage, but instead PLAC1 expression and actions were linked to syncytiotrophoblast differentiation. Furthermore, the impact of PLAC1 on cellular function is linked to furin (paired basic amino acid cleaving enzyme) in rat and human trophoblast cells. Thus, PLAC1 plays an important role in hemochorial placentation; however, the responsive trophoblast cell lineages and its contributions to placentation are fundamentally distinct in the rat versus human.

## Teaser

**Highlighted Article:** PLAC1 plays distinct roles in rat and human placentation, influencing different trophoblast lineages in each species.

## INTRODUCTION

The placenta is an extra-embryonic structure that develops along with the embryo, ensuring the survival and development of the fetus within the female reproductive tract (Burton et al., 2016; Hemberger and Dean, 2023; Maltepe and Fisher, 2015). Trophoblast cells are specialized cell types of the placenta responsible for executing critical functions. These functions include modification of the maternal environment through hormone production and transformation of the uterine vasculature and by acting as a barrier regulating the delivery of compounds to the fetal vasculature (Hemberger et al., 2020; Knöfler et al., 2019; Soares et al., 2018; Turco and Moffett, 2019). Failures in placentation are directly linked to obstetrical complications, including disorders such as pre-eclampsia, intrauterine growth restriction, and preterm birth (Brosens et al., 2019, 2011; Burton et al., 2016; Maltepe and Fisher, 2015). There is a paucity of knowledge regarding the molecular mechanisms controlling placentation.

The X chromosome has been directly linked to pregnancy success (Hemberger, 2002). X chromosome dosage is relevant to normal cellular function necessitating inactivation of one of the X chromosomes (Lee and Bartolomei, 2013). In rodents, the paternal X chromosome is preferentially inactivated in extra-embryonic tissues, including the placenta (Frels and Chapman, 1980; Harper et al., 1982; Takagi and Sasaki, 1975; West et al., 1977). Several studies have associated the X chromosome, and more specifically X chromosome-linked genes, with the regulation of placentation (Hemberger, 2002; Hemberger et al., 1999; Kushi et al., 1998; Marahrens et al., 1997; Shao and Takagi, 1990; Surani and Barton, 1983; Zechner et al., 1997, 1996). Among the X-linked genes, placenta enriched 1 (*Plac1*) is a poorly understood regulator of placentation (Fant et al., 2010).

The *PLAC1* gene is conserved throughout placental mammals (Devor, 2014). Expression profiling has demonstrated the selective and abundant expression of PLAC1 in the placenta (Cocchia et al., 2000). PLAC1 expression is increased following *in vitro* differentiation of rodent and human trophoblast cells (Chang et al., 2014; Donnison et al., 2015; Gu et al., 2017; Kent et al., 2010; Massabbal et al., 2005). *In vitro* experiments have also implicated PLAC1 as a regulator of trophoblast cell differentiation, including impacts on endocrine, invasive and syncytiotrophoblast (STB) cell lineages (Chang et al., 2016, 2014; Gu et al., 2017). Mouse mutagenesis has provided some insights into the biology of PLAC1 in placentation (Jackman et al., 2012; Muto et al., 2016). Disruptions of the mouse *Plac1* locus result in placentomegaly (Jackman et al., 2012; Muto et al., 2016).

The cellular actions of PLAC1 are not well understood. The predicted PLAC1 protein structure resembles a membrane protein with a short N-terminal intracellular region, a transmembrane segment, and a longer extracellular domain resembling a truncated version of a zona pellucida domain (Mahmoudian et al., 2019). PLAC1 has been localized to the plasma membrane, cytoplasm and nucleus (Fant et al., 2010; Mahmoudian et al., 2019). Several cancers, including breast, colorectal and prostate, produce PLAC1, where it contributes to the regulation of cell proliferation, adhesion and metastasis (Fant et al., 2010; Mahmoudian et al., 2019). Furthermore, PLAC1 has been linked to the regulation of phoshoinositide-3-kinase/AKT signaling (Ma et al., 2021; Roldán et al., 2020; Wu et al., 2017), and interacts with other cellular proteins (Chen et al., 2021). Some PLAC1–protein interactions may occur via its zona pellucida domain (Jovine et al., 2006).

In this study, we explore the involvement of PLAC1 in the regulation of trophoblast cell development and deep placentation *in vivo*, using a genetically manipulated rat model, and *in vitro* using rat and human trophoblast stem cells (TSCs). The rat is used to investigate the biology of placentation because it exhibits deep trophoblast cell invasion and extensive trophoblast-guided restructuring of the uterine vasculature as is observed in human placentation (Ain et al., 2003; Pijnenborg et al., 1981; Pijnenborg and Vercruysse, 2010; Shukla and Soares, 2022; Soares et al., 2012). Herein, we demonstrate that PLAC1 plays prominent roles in rat and human trophoblast cell development; however, the nature of PLAC1 actions has striking elements of species specificity.

## RESULTS

### PLAC1 expression within the rat placentation site

**Fig. 1.**
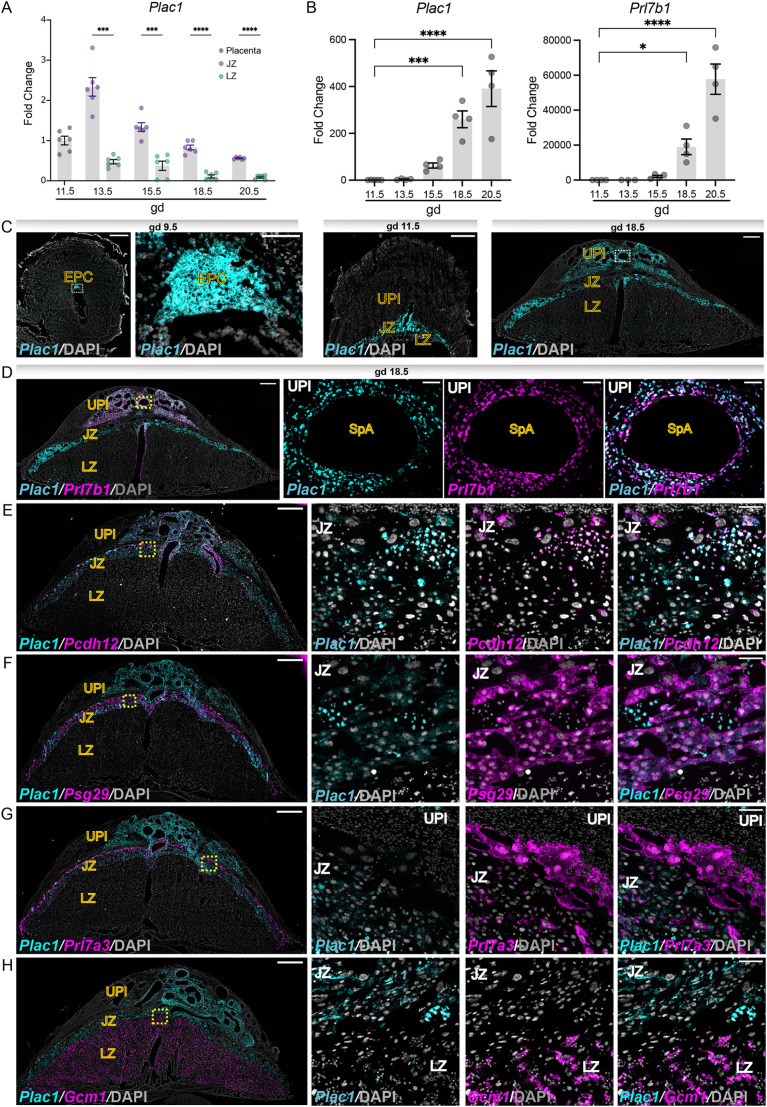
**Expression of *Plac1* transcripts within the placentation site during the second half of gestation in the rat.** (A) RT-qPCR measurements of *Plac1* transcript levels in junctional zone and labyrinth zone compartments of the rat placenta across different gestational stages. *Gapdh* was used to normalize the measurements. Data are presented as mean±s.e.m. Dots represent biological replicates per condition (*n*=6). ***P<0.0005, ****P<0.0001 (Brown–Forsythe and Welch analysis of variance tests). (B) *Prl7b1* and *Plac1* transcript expression in rat uterine–placental interface tissue examined by RT-qPCR during the second half of gestation. Data are presented as mean±s.e.m. Dots represent biological replicates per condition (*n*=4). *P<0.05, ***P<0.0005, ****P<0.0001 (one-way analysis of variance and Dunnett's multiple comparisons test). (C) Representative images of *Plac1* (cyan) *in situ* hybridization at gd 9.5, 11.5 and 18.5. Dashed white boxes (left and right panels) are shown at higher magnification in the respective panels (middle). (D-H) Representative images of *in situ* hybridization for *Plac1* (cyan) and *Prl7b1*, *Pcdh12*, *Psg29*, *Prl7a3* or *Gcm1* (magenta), respectively, in placentation sites at gd 18.5. Dashed yellow boxes in the left panels are shown at higher magnification on the right. Images are representative of 3 samples. EPC, ectoplacental cone; JZ, junctional zone; LZ, labyrinth zone; SpA, spiral artery; UPI, uterine–placental interface. Scale bars: 50 μm (C, gd 9.5 high-magnification image); 100 μm (D-H, high-magnification images); 1000 μm (all other panels).

Earlier reports in the mouse and human demonstrated placental-specific expression of PLAC1 with higher expression in the midgestation mouse placenta, whereas PLAC1 was expressed at similar levels throughout gestation in the human (Cocchia et al., 2000; Fant et al., 2002; Gu et al., 2017). Initially, *Plac1* transcripts were estimated within compartments of the rat placentation site (junctional zone, labyrinth zone and uterine–placental interface) by reverse transcription-quantitative polymerase chain reaction (RT-qPCR). *Plac1* transcripts were prominently expressed in the junctional zone of the placentation site across all gestational stages but not in the labyrinth zone (Fig. 1A). The rat uterine–placental interface exhibited progressively increased expression of *Plac1* transcripts as gestation advanced, showing maximal expression on gestation day (gd) 20.5 (Fig. 1B), which parallels the expansion of invasive trophoblast cells into the uterine parenchyma (Ain et al., 2003) and the increased expression of invasive trophoblast cell-specific transcripts, including prolactin family 7, subfamily b, member 1 (*Prl7b1*; Fig. 1B). The decrease in *Plac1* transcript expression in the junctional zone during late gestation (Fig. 1A) may reflect the differentiation of junctional zone-derived invasive trophoblast cells that migrated into the uterine–placental interface. This observation is consistent with a progressive enrichment of *Plac1^+^* invasive trophoblast cells within the uterine–placental interface as gestation advances (Fig. 1B).

*Plac1* transcripts were specifically localized to trophoblast cell lineages, as demonstrated by *in situ* hybridization. At gd 9.5, an early stage of placentation, *Plac1* transcripts were specifically localized to the ectoplacental cone (Fig. 1C), which gives rise to the junctional zone. As gestation advanced, the junctional zone was positive for *Plac1* transcript expression, whereas *Plac1* expression in the labyrinth zone was not detectable (Fig. 1C). During the last week of gestation, *Plac1* expression extended to the invasive trophoblast cell population of the uterine–placental interface, and colocalized with *Prl7b1*, an invasive trophoblast cell-specific transcript (Wiemers et al., 2003) (Fig. 1C). Single-cell RNA sequencing (scRNA-seq) of the rat uterine–placental interface also demonstrated the expression of *Plac1* transcripts in invasive trophoblast cells (Scott et al., 2022; Vu et al., 2023; Fig. S1). Two distinctive populations of invasive trophoblast cells exist: endovascular and interstitial (Shukla and Soares, 2022; Soares et al., 2012). Endovascular invasive trophoblast cells target uterine spiral arteries, enter the vessels, and replace the endothelium, whereas interstitial invasive trophoblast cells migrate through the uterine stroma. These two invasive trophoblast cell populations coordinate the transformation of the uterine vasculature. *Plac1* expression within the uterine–placental interface was primarily restricted to interstitial invasive trophoblast cells (Fig. 1D). Within the junctional zone, *Plac1* expression colocalized predominantly with glycogen cells (*Pcdh12* positive; Fig. 1E) and to a lesser extent with spongiotrophoblast cells (*Psg29* positive; Fig. 1F). *Plac1* expression was not detected in trophoblast giant cells (*Prl7a3* positive; Fig. 1G) or in the labyrinth zone (*Gcm1* positive; Fig. 1H). Collectively, our observations place PLAC1 within trophoblast cell populations directly linked to the establishment of the uterine–placental interface.

### PLAC1 modulates placental size

**Fig. 2.**
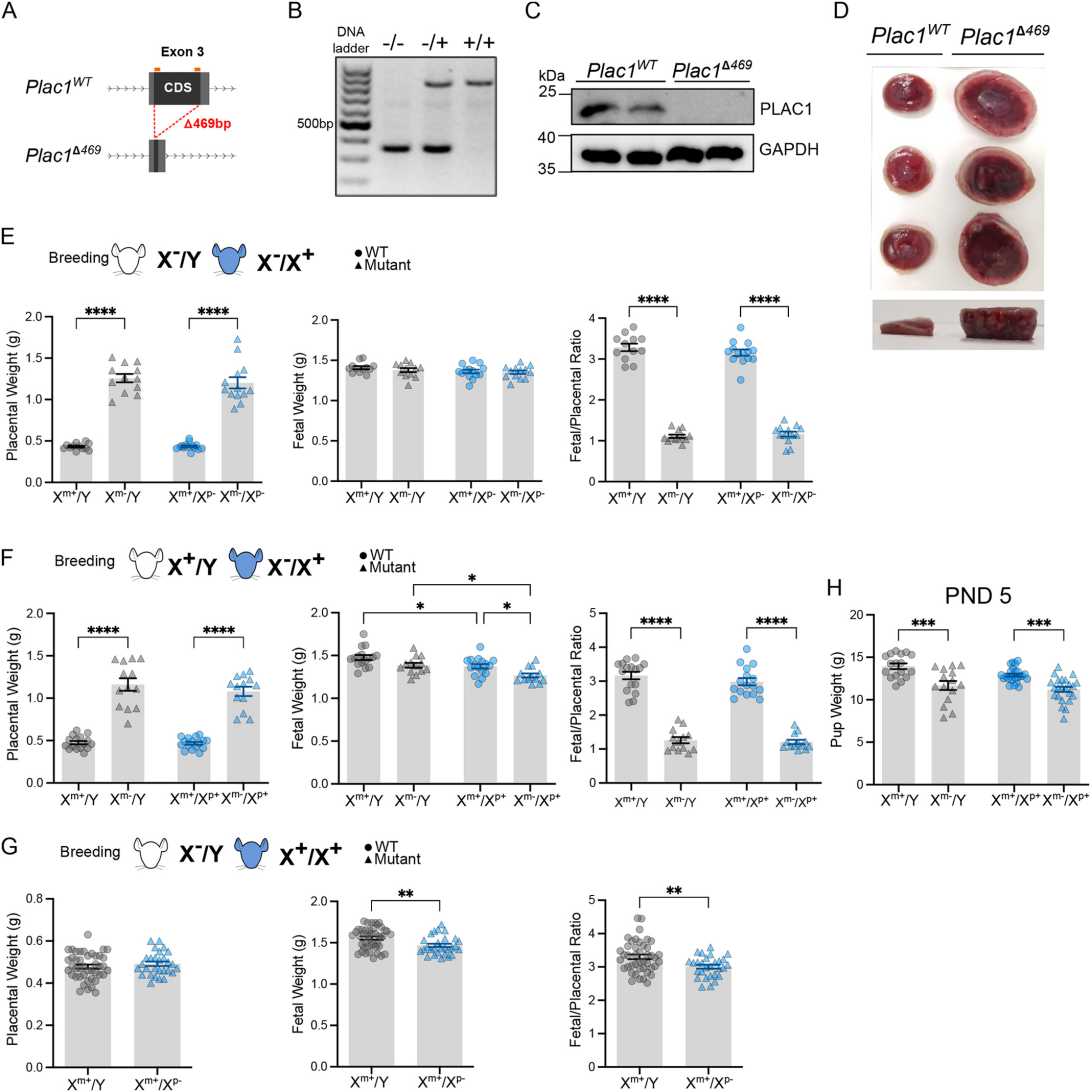
**PLAC1 actions on placental and fetal development are dependent upon the parental origin of the X chromosome.** (A) Schematic of the wild-type (*Plac1^WT^*) and *Plac1* mutant (*Plac1*^Δ*469*^) alleles used for generation of the *Plac1* mutant rat. CRISPR/Cas9 targeting of exon 3 of the rat *Plac1* gene yielded a 469 bp deletion. CDS, coding DNA sequence. (B) Genotyping of offspring for wild-type and *Plac1* mutant alleles. (C) Western blot analysis of wild-type versus *Plac1* mutant gd 18.5 junctional zone tissues. *n*=2. (D) Gross inspection of wild-type and *Plac1* mutant placentas at gd 20.5. (E-G) Schematics of breeding strategies are shown for each panel. (E) Effect of mutant *Plac1* allele on placental and fetal weights and placenta efficiency (fetal/placental weight ratio) on gd 18.5 (*n*=5 litters, 54 conceptuses). (F) Effects of a maternally inherited mutant *Plac1* allele (X^m−^) on placental and fetal weights and placenta efficiency on gd 18.5 (*n*=5 litters, 58 conceptuses). (G) Effects of a paternally inherited mutant *Plac1* allele (X^p−^) on placental and fetal weights and placenta efficiency on gd 18.5 (*n*=5 litters, 71 conceptuses). (H) Effects of a maternally inherited mutant *Plac1* allele (X^m−^) on pup weights on postnatal day 5 (PND5; *n*=6 litters, 80 pups). Triangles represent mutants, circles represent wild type. Gray: males; blue: females. Data are presented as mean±s.e.m. Each dot represents a biological replicate. *P<0.05, **P<0.005, ***P<0.0005, ****P<0.0001 (for E,F, one-way analysis of variance and Holm–Šidák multiple comparison test; for G, unpaired *t*-test).

To study the role of PLAC1 in pregnancy and placentation we used a loss-of-function approach. The *Plac1* locus was mutated using CRISPR/Cas9 genome editing. The PLAC1 coding exon was targeted to produce a 469 bp deletion (Fig. 2A). The deletion was detected by PCR (Fig. 2B) and confirmed by DNA sequencing, which indicated that nucleotides coding for >95% of the predicted PLAC1 amino acid sequence were removed. The disruption of PLAC1 protein in *Plac1* mutant placentas was demonstrated by western blotting (Fig. 2C). Homozygous *Plac1* mutant female rats (*Plac1^X−/X−^*) and hemizygous mutant male rats (*Plac1^X−/Y^*) exhibited postnatal weight gain similarly to wild-type rats and were fertile.

We next examined the consequences of *Plac1* gene disruption on placental and fetal development. *Plac1^X−/X+^* female rats were mated to *Plac1^X−/Y^* male rats. Females were euthanized on gd 13.5, 15.5, 18.5 and 20.5. Genotyping of fetuses confirmed the expected Mendelian ratio. Half of the female fetuses and all the male fetuses with a mutant *Plac1* allele had placentas significantly larger than placentas associated with wild-type fetuses (Fig. 2D,E). The magnitude of the placentomegaly increased as gestation advanced (Fig. S2). Fetal weights were similar among all genotypes except on gd 20.5, when half of the female fetuses and all male fetuses with a mutant *Plac1* allele were significantly smaller than wild-type fetuses (Fig. 2E, Fig. S2). Fetal/placental weight ratios were significantly smaller in *Plac1* mutant conceptuses versus wild-type conceptuses, indicating that *Plac1* mutant placentas exhibited features of inefficiency (Fig. 2E).

In summary, disruption of the *Plac1* gene in the rat leads to the development of a very large and inefficient placenta with a modest impact on fetal growth.

### Mutant *Plac1* phenotype depends on the origin of the X chromosome

The *Plac1* locus is situated on the X chromosome (Cocchia et al., 2000; Hemberger et al., 2000). Expression of X-linked genes in mouse trophoblast cells is influenced by the preferential inactivation of the paternal X chromosome (Frels and Chapman, 1980; Harper et al., 1982; Takagi and Sasaki, 1975; West et al., 1977). Thus, the origin of the X chromosome could impact phenotypes exhibited by *Plac1* heterozygous X,X placentas. The expectation is that inheritance of a maternal X chromosome with a mutant *Plac1* allele would exhibit a phenotype similar to that of a *Plac1* null; whereas inheritance of a paternal X chromosome with a mutant *Plac1* allele would exhibit a phenotype similar to a wild-type conceptus. We mated heterozygous females with wild-type males and wild-type females with *Plac1* hemizygous males. Indeed, inheritance of the *Plac1* mutant allele from the mother resulted in placentomegaly, whereas inheritance of the *Plac1* mutant allele from the father resulted in a normal placental phenotype (Fig. 2F,G). Placenta efficiency (fetus/placenta ratio) was also decreased in placentas possessing a maternally inherited *Plac1* mutant allele (Fig. 2F). Interestingly, we observed significant growth restriction at postnatal day 5 in animals possessing a maternally inherited *Plac1* mutant allele (Fig. 2H). The experimentation is consistent with preferential inactivation of the paternal X chromosome in trophoblast cells and the importance of the maternal X chromosome in placentation.

### PLAC1 restrains junctional zone growth

**Fig. 3.**
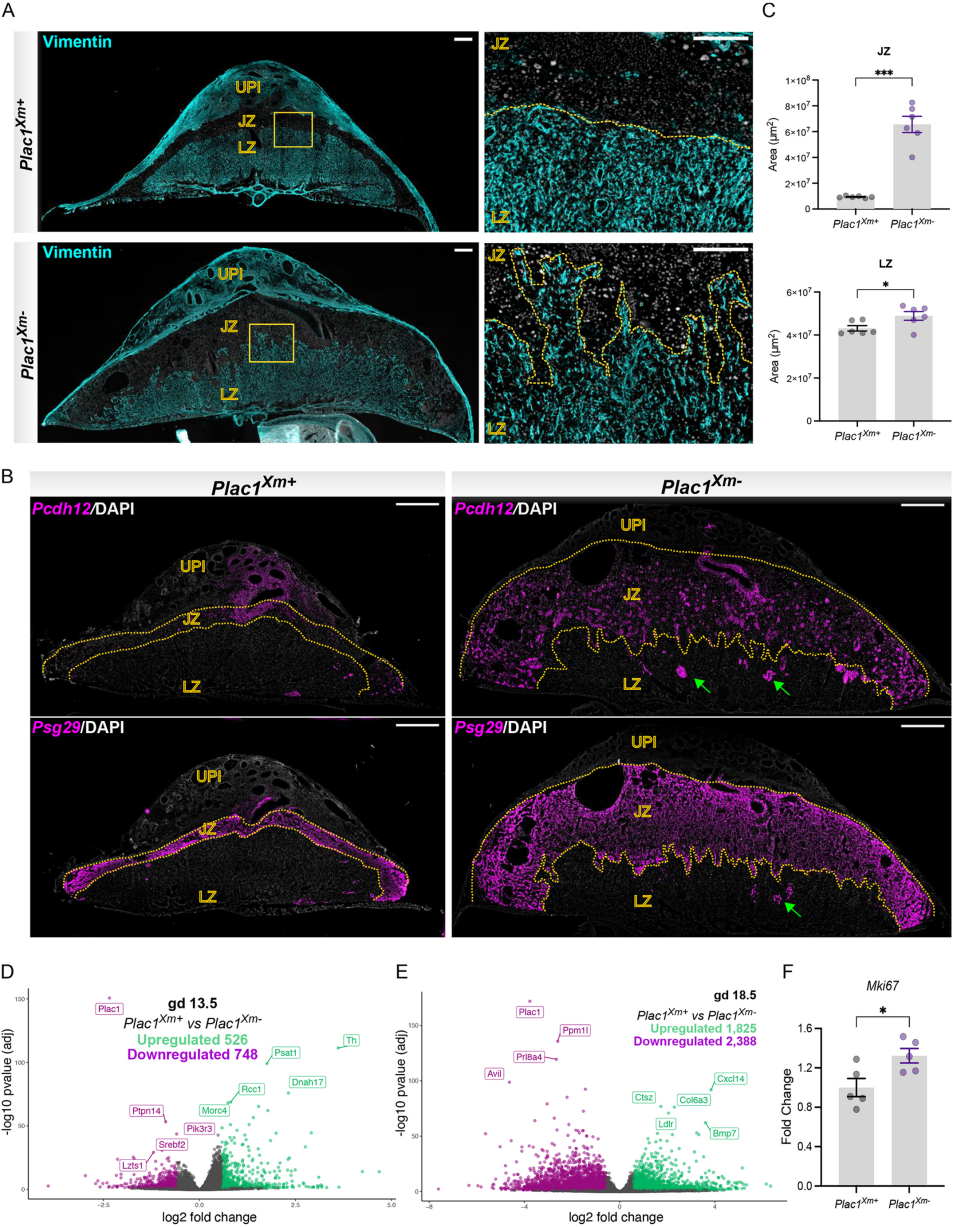
**Loss of PLAC1 results in disruptions in the integrity of placental compartments.** (A) Representative images of vimentin immunostaining of wild-type (*Plac1^Xm+^*) and *Plac1* mutant (*Plac1^Xm−^*) placentation sites at gd 18.5. Yellow boxes in the left panels are shown at higher magnification in the respective right panels. (B) *Pcdh12* and *Psg29* transcript localization within wild-type (*Plac1^Xm+^*) and *Plac1* mutant (*Plac1^Xm−^*) placentation sites at gd 18.5. Green arrows indicate the location of *Pcdh12*-positive and *Psg29*-positive cell clusters within the labyrinth zone. (C) Area measurements of junctional zone and labyrinth zone in *Plac1^Xm+^* and *Plac1^Xm−^* placentas. Data are presented as mean±s.e.m. Dots represent biological replicates per condition (*n*=6). ***P<0.0005 (unpaired *t*-test). Dotted lines in A indicate the junctional zone-labyrinth zone interface; dotted lines in B delineate the boundary of the junctional zone. (D,E) Volcano plot of RNA-seq analysis of *Plac1^Xm−^* versus *Plac1^Xm+^* of placentas at gd 13.5 and junctional zone at gd 18.5, respectively. The most prominent DEGs are highlighted. (F) RT-qPCR measurement of *Mki67* in *Plac1^Xm+^* and *Plac1^Xm−^* junctional zone at gd 18.5. Data are presented as mean±s.e.m. Each dot represents a biological replicate (*n*=5-6). *P<0.05, ***P<0.0005 (unpaired *t*-tests). JZ, junctional zone; LZ, labyrinth zone; UPI, uterine–placental interface. Scale bars: 1000 μm.

The rat placenta is organized into two compartments: the junctional zone and the labyrinth zone (Soares et al., 2012). The junctional zone is situated at the uterine interface, whereas the labyrinth zone directly connects to the developing fetus. We next sought to determine how *Plac1* mutant-associated placentomegaly impacted development of each placental compartment. We used vimentin immunostaining to distinguish between junctional and labyrinth zones at gd 18.5 in wild-type (*Plac1^Xm+^*) and mutant (*Plac1^Xm−^*) placentas. Vimentin is present in the mesenchymal/stromal components of the labyrinth zone and the uterus, whereas the junctional zone is devoid of vimentin immunostaining (Konno et al., 2007). We observed three prominent features characteristic of *Plac1* mutant placentas: (1) expansion of the junctional zone (Fig. 3A); (2) an irregular junctional zone–labyrinth zone interface (Fig. 3A); and (3) clusters of glycogen cells (*Pcdh12* positive) and spongiotrophoblast cells (*Psg29* positive) within the labyrinth zone compartment (Fig. 3B). Expansion of the junctional zone in *Plac1* mutants (*Plac1^Xm−^*) included contributions from both glycogen cells (*Pcdh12* positive) and spongiotrophoblast (*Psg29* positive) cellular constituents (Fig. 3B). We further quantified the areas of each zone from wild-type and mutant placentas (Fig. 3C). The increase in the size of the *Plac1^Xm−^* mutant placenta is primarily associated with an increase in the size of the junctional zone. The junctional zone contains trophoblast cell lineages possessing endocrine, metabolic and invasive properties (Shukla and Soares, 2022). Monocarboxylate transporter 1 (MCT1; SLC16A1) and monocarboxylate transporter 4 (MCT4; SLC16A3) proteins can be effectively used to assess labyrinth zone integrity (Nagai et al., 2010). MCT1 and MCT4 immunostaining revealed no apparent disruption of labyrinth zone architecture in *Plac1* mutant (*Plac1^Xm−^*) placentas (Fig. S3A). The quantification of staining intensity showed no difference in MCT1 and a modest reduction of MCT4 in *Plac1* mutant (*Plac1^Xm−^*) placentas.

### Transcript profiles in wild-type and PLAC1-deficient placentas

To gain additional insight into the role of PLAC1 in placental development, we performed RNA sequencing (RNA-seq) on gd 13.5 placental tissue and gd 18.5 junctional zone tissue in wild-type and PLAC1-deficient placentas. At gd 13.5, the placental compartments are well defined and the uterine–placental interface exhibits limited intrauterine trophoblast cell invasion, whereas the gd 18.5 is characterized by well-defined placental compartments and maximal intrauterine trophoblast cell invasion. Transcripts with a fold-change of ≥1.5 and an adjusted *P*-value of <0.05 were considered differentially regulated. Striking differences in gene expression were noted between wild-type and PLAC1-deficient placental tissues (Fig. 3D,E).

At gd 13.5, RNA-seq identified 1274 differentially regulated transcripts, including 748 downregulated transcripts and 526 upregulated transcripts in PLAC1-deficient placental tissues (Fig. 3D, Table S1). Representative downregulated and upregulated transcripts were validated by RT-qPCR (Fig. S4A). Kyoto Encyclopedia of Genes and Genomes (KEGG) enrichment analysis and Gene Set Enrichment Analysis (GSEA) highlighted a downregulation of Janus kinase-signal transducer and activator of transcription and focal adhesion pathways and an upregulation of the peroxisome proliferator-activated receptor gamma pathway and transcripts associated with lipid metabolism (Fig. S5).

At gd 18.5, RNA-seq identified 4213 differentially regulated transcripts, including 2388 downregulated transcripts and 1825 upregulated transcripts in PLAC1-deficient junctional zone tissue (Fig. 3E, Table S2). Representative downregulated and upregulated transcripts were validated by RT-qPCR (Fig. S4B). KEGG and GSEA enrichment analyses highlighted a downregulation of transcripts associated with endopeptidase activity and WNT signaling, and an upregulation of transcripts associated with the cell cycle (Fig. S6). The latter was exemplified by an increase in the expression of marker of proliferation Ki-67 (*Mki67*) transcript within the gd 18.5 *Plac1^Xm−^* junctional zone (Fig. 3F) and an increase in the number of MKI67 protein-positive cells within the gd 18.5 *Plac1^Xm−^* junctional zone (Fig. S7). These observations related to MKI67 expression reflect increased cell proliferation and offer a potential explanation for the expansion of the junctional zone associated with PLAC1 deficiency.

We next used a mouse placental single-nucleus RNA-seq dataset (Marsh and Blelloch, 2020) to provide insight into junctional zone cell types contributing to the transcriptomic disruptions accompanying PLAC1 deficiency. At gd 13.5, transcripts associated with spongiotrophoblast progenitor cells, spongiotrophoblast cells and glycogen cells were downregulated (Fig. S8A). As gestation proceeded (gd 18.5), transcripts associated with junctional zone progenitor cells and glycogen cells were upregulated, whereas transcripts associated with spongiotrophoblast were downregulated (Fig. S8B).

In summary, the striking effects of PLAC1 deficiency on the organization of the rat hemochorial placenta are reinforced by dramatic differences in transcript profiles.

### PLAC1 promotes intrauterine trophoblast cell invasion

**Fig. 4.**
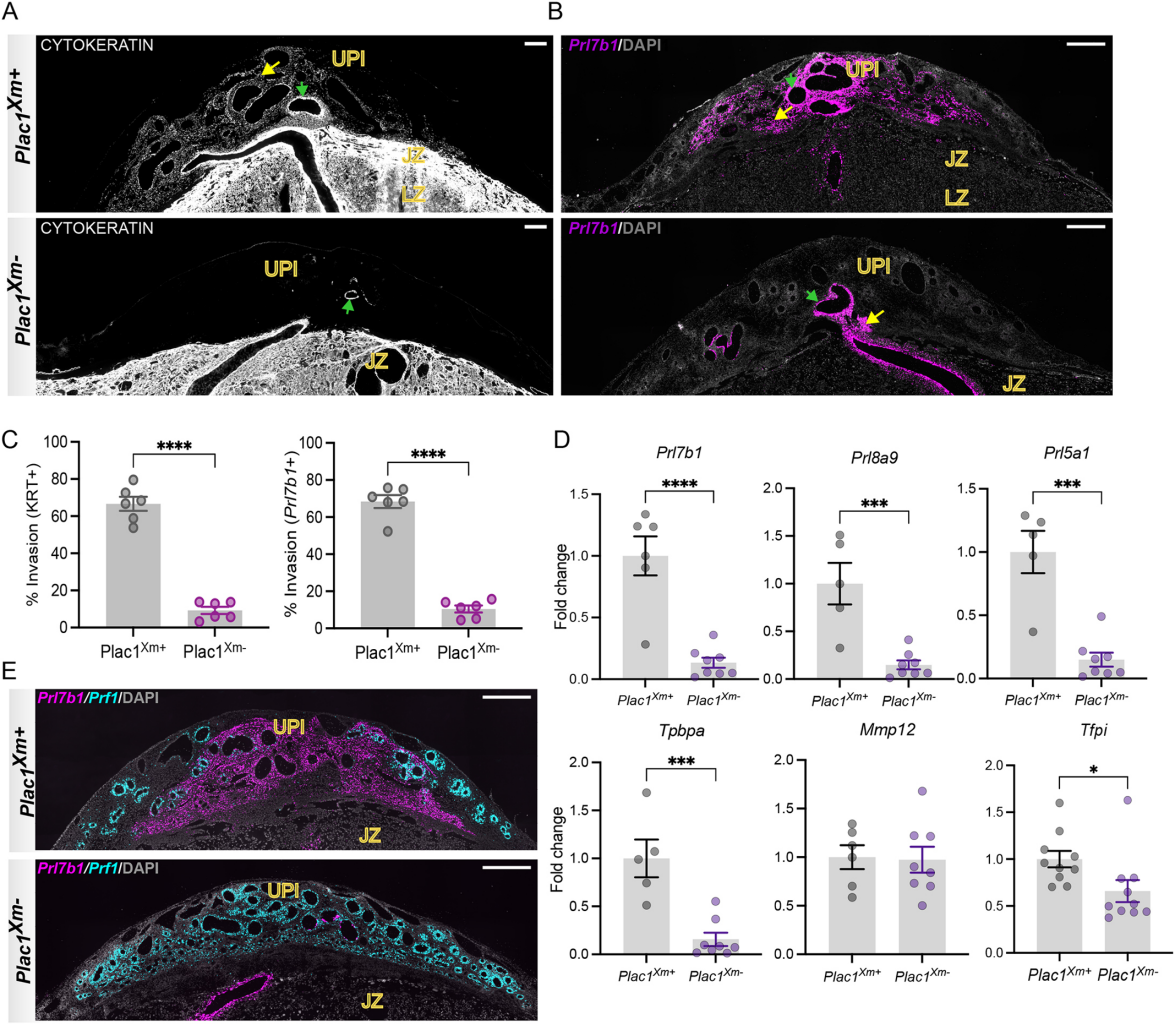
**PLAC1 deficiency is associated with deficits in intrauterine trophoblast cell invasion.** (A) Representative images of cytokeratin immunostaining of wild-type (*Plac1^Xm+^*) and *Plac1* mutant (*Plac1^Xm−^*) placentation sites at gd 18.5. (B) Representative images of *Prl7b1 in situ* hybridization of wild type (*Plac1^Xm+^*) and *Plac1* mutant (*Plac1^Xm−^*) placentation sites at gd 18.5. In A and B, the locations of representative interstitial invasive trophoblast cells are demarcated by yellow arrows and representative endovascular invasive trophoblast cells are demarcated by green arrows. (C) Quantification of total invasion in the uterine–placental interface as detected by cytokeratin (KRT) immunostaining (left) and by *Prl7b1 in situ* hybridization (right). Data are presented as mean±s.e.m. Each dot represents a biological replicate (*n*=6). ****P<0.0001 (unpaired *t*-test). (D) RT-qPCR measurements of invasive trophoblast cell-associated transcripts in *Plac1^Xm+^* and *Plac1^Xm−^* gd 18.5 uterine–placental interface tissue specimens. Data are presented as mean±s.e.m. Each dot represents a biological replicate (*n*=5-8). *P<0.05, ***P<0.0005, ****P<0.0001 (unpaired *t*-tests). (E) Representative images of *Prl7b1* and *Prf1 in situ* hybridization of wild-type (*Plac1^Xm+^*) and *Plac1* mutant (*Plac1^Xm−^*) placentation sites at gd 18.5. JZ, junctional zone; LZ, labyrinth zone; UPI, uterine–placental interface. Scale bars: 500 μm (A); 1000 μm (B,E).

The junctional zone is the source of progenitor cells that differentiate into invasive trophoblast cells, which transit into and transform the uterus (Soares et al., 2012). *Plac1* is prominently expressed in both the junctional zone and in invasive trophoblast cells (Fig. 1) and is a major regulator directing junctional zone development (Figs 2 and 3). Consequently, we examined the impact of PLAC1 deficiency on invasive trophoblast cell infiltration into the uterus. Cytokeratin immunostaining recognizes the epithelial character of trophoblast cells and was used to visualize invasive trophoblast cells at the uterine–placental interface (Ain et al., 2003). Endovascular trophoblast cells were present at the uterine–placental interface of both wild-type (*Plac1^Xm+^*) and *Plac1* mutant (*Plac1^Xm−^*) placentation sites (Fig. 4A). In contrast, interstitial trophoblast cells were depleted from the uterine–placental interface of *Plac1* mutant (*Plac1^Xm−^*) placentation sites (Fig. 4A). Similar distributions were observed when invasive trophoblast cells were monitored by *in situ* hybridization for *Prl7b1* in wild-type and *Plac1* mutant (*Plac1^Xm−^*) placentation sites (Fig. 4B). The surface area within the uterine–placental interface occupied by invasive trophoblast cells was significantly decreased in *Plac1* mutant (*Plac1^Xm−^*) placentation sites (Fig. 4C). Comparable outcomes were noted in placentation sites associated with female (*Plac1^Xm−/Xp+^* and *Plac1^Xm−/Xp−^*) and male (*Plac1^Xm−/Y^*) fetuses. To explore these PLAC1-dependent actions further, we examined the expression of additional transcripts specific to invasive trophoblast cells within the uterine–placental interface of wild type and *Plac1* mutants. *Mmp12* and *Tfpi* transcripts are enriched in endovascular invasive trophoblast cells (Chakraborty et al., 2016; Muto et al., 2021) and exhibited no or modest changes in wild-type versus PLAC1-deficient uterine–placental interface tissue (Fig. 4D). In contrast, other invasive trophoblast cell-specific transcripts (*Prl7b1, Prl5a1, Prl8a9, Tpbpa*; Scott et al., 2022) were dramatically downregulated in the uterine–placental interface from PLAC1-deficient placentation sites (Fig. 4D). Interestingly, the uterine–placental interface from late-gestation-stage PLAC1-deficient placentation sites exhibited a prominent infiltration of perforin positive natural killer (NK) cells (Fig. 4E), a phenomenon previously reported in another mutant rat model exhibiting impaired intrauterine trophoblast cell invasion (Dominguez et al., 2025).

### PLAC1 regulates rat trophoblast cell differentiation

We next investigated the biology of PLAC1 in rat trophoblast cell differentiation. Rat TSCs represent a useful *in vitro* model for investigating junctional zone development (Asanoma et al., 2011; Chakraborty et al., 2016, 2011; Kubota et al., 2015; Muto et al., 2021; Varberg et al., 2021). PLAC1 transcript and protein levels increased throughout the 15-day time course of trophoblast cell differentiation (Fig. S9B,C), which positively correlated with the expression profile of *Prl7b1* (Fig. S9B), a transcript associated with acquisition of an invasive trophoblast cell phenotype (Wiemers et al., 2003). We next used a loss-of-function approach to investigate the involvement of PLAC1 in rat TSC differentiation. Control and *Plac1* short hairpin RNAs (shRNAs) were delivered to rat TSCs in the stem state by lentiviral-mediated transduction. Disruption of *Plac1* transcript and protein expression were confirmed in cells containing *Plac1* shRNA (Fig. S9D). PLAC1 disrupted TSCs exposed to conditions promoting differentiation failed to exhibit morphological features of differentiation but instead exhibited features resembling TSCs in the stem state (Fig. S9A,E). Transcriptomes of differentiated rat TSCs expressing control or *Plac1* shRNAs were interrogated by RNA-seq. Depletion of PLAC1 led to 4472 differentially regulated transcripts, including 1933 downregulated transcripts and 2539 upregulated transcripts (Fig. S9F, Table S3). Many of the downregulated genes were associated with differentiation-induced transcripts (Fig. S9G; Muto et al., 2021) and several upregulated genes were TSC stem state-associated transcripts (Fig. S9G; Muto et al., 2021). Representative downregulated and upregulated transcripts were validated by RT-qPCR (Fig. S9H). *Mki67* was upregulated in PLAC1-depleted rat TSCs after differentiation (Fig. S9I), as previously observed in the PLAC1-deficient gd 18.5 placenta (Fig. 3E). Depletion of PLAC1 in the TSC stem state did not significantly affect cell proliferation (Fig. S9J). KEGG and GSEA enrichment analyses paralleled findings in control and PLAC1-deficient rat placentas (Figs S5, S6 and S10). Linkage of PLAC1-responsive differentially expressed genes (DEGs) in rat TSCs to junctional zone cell types using the mouse placental single-nucleus RNA-seq dataset (Marsh and Blelloch, 2020) revealed a pattern similar to that observed in gd 13.5 rat placenta (Figs S8A and S11). We observed a cellular phenotype reflecting and upregulation of transcripts associated with junctional zone progenitor cells and downregulation of transcripts associated with spongiotrophoblast progenitor cells, spongiotrophoblast cells and glycogen cells (Fig. S11).

In summary, PLAC1-deficient TSCs exposed to differentiating conditions fail to differentiate and exhibit stem state character.

### PLAC1 in the human placenta

Roles for PLAC1 in human trophoblast cell function have previously been reported (Chang et al., 2016, 2014; Massabbal et al., 2005), suggesting that some aspects of PLAC1 action in placental development could be conserved. However, existing reported experimentation is limited and potentially contradictory.

**Fig. 5.**
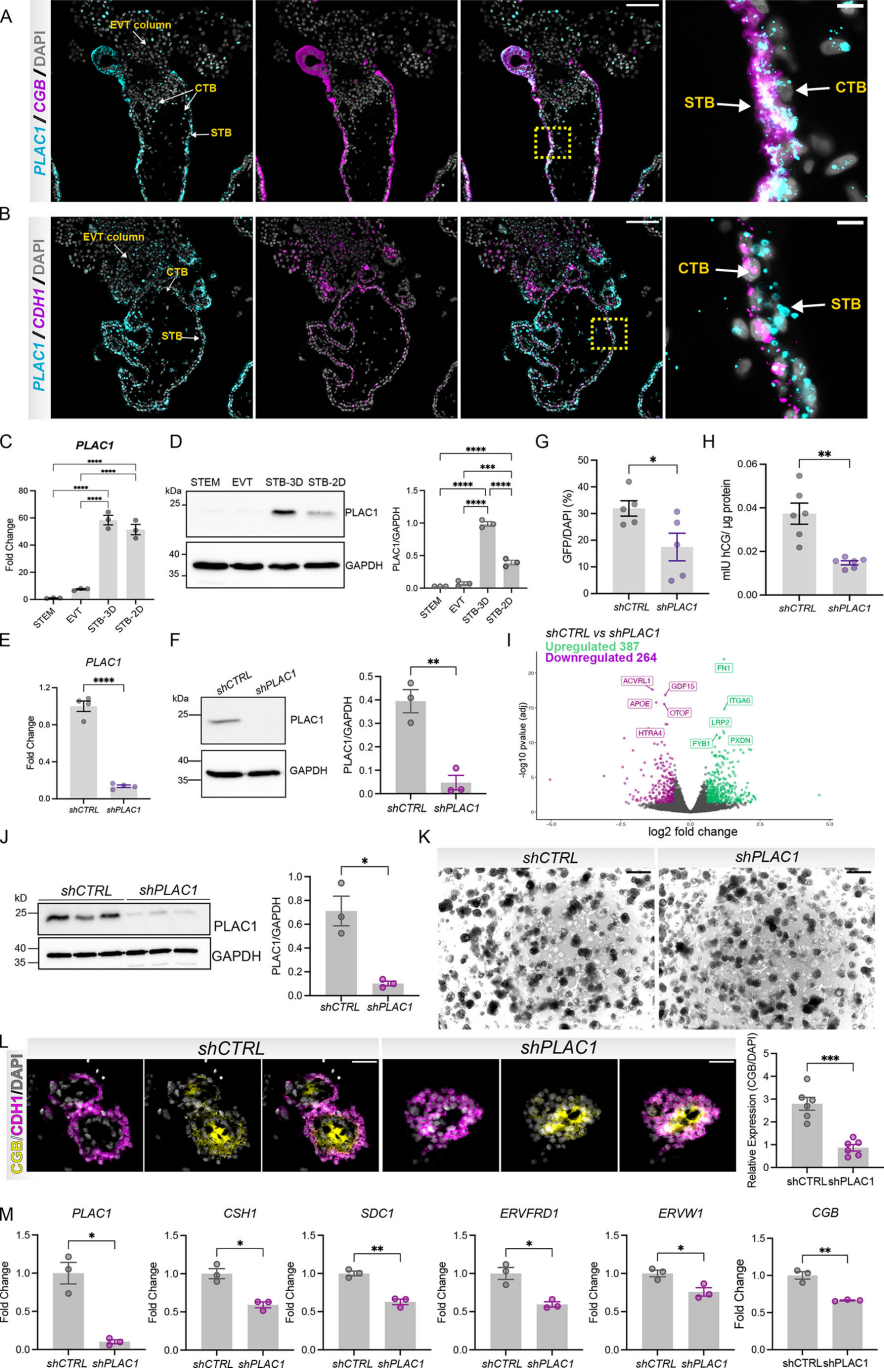
**PLAC1 in the human placenta and human TSCs (CT-27).** (A) Representative images of *PLAC1* (cyan) and *CGB* (magenta) transcripts detected by *in situ* hybridization in tissue sections from the first trimester human placenta (11 weeks). (B) Representative images of *PLAC1* (cyan) and *CDH1* (magenta) transcripts detected by *in situ* hybridization in tissue sections from the first trimester human placenta. In A,B, dashed yellow boxes are shown at higher magnification in the respective right panels. (C) RT-qPCR measurements of *PLAC1* transcripts in human TSCs in the stem cell state and following EVT cell or STB differentiation. *POLR2A* was used to normalize the measurements. (D) Representative PLAC1 western blot and quantification relative to GAPDH for human TSCs in the stem cell state and following EVT cell and STB differentiation. (E,F) RT-qPCR and western blot analyses of control shRNA (*shCTRL*)- and PLAC1 shRNA (*shPLAC1*)-treated human TSCs in the STB-3D differentiation state. (G) Cell fusion assessment of *shCTRL-* and *shPLAC1*-treated human TSCs in the STB-2D differentiated state. (H) CG enzyme-linked immunoassay measurements of *shCTRL*- and *shPLAC1*-treated human TSCs in the STB-2D differentiated state. (I) Volcano plot of RNA-seq analysis of *shCTRL-* and *shPLAC1*-treated human TSCs in the STB-3D differentiation state. The most prominent DEGs are highlighted. (J) Western blot analyses of *shCTRL-* and *shPLAC1*-treated human TOs and associated quantification. PLAC1 protein was normalized to GAPDH (right). (K) Representative phase-contrast images of *shCTRL-* and *shPLAC1*-treated TSC-derived trophoblast organoids (TOs). (L) Representative images of CGB and CDH1 immunostaining of *shCTRL*- and *shPLAC1*-treated TOs and associated quantification. (M) RT-qPCR measurements of STB associated transcripts in *shCTRL-* and *shPLAC1*-treated TOs. Data are presented as mean±s.e.m. Each dot represents a biological replicate (*n*=3-6). *P<0.05, **P<0.005, ***P<0.0005, ****P<0.0001 (for C,D, one-way analysis of variance and Tukey's multiple comparisons test; for E-H,J,L,M, unpaired *t*-test). CTB, cytotrophoblast; EVT, extravillous trophoblast; STB, syncytiotrophoblast*.* Scale bars: 50 μm (A, left three panels; B, left three panels; L); 10 μm (A, right panel; B, right panel); 500 μm (K).

We first examined *PLAC1* expression in human placental tissue by *in situ* hybridization. *PLAC1* transcripts were localized to STB of first trimester human placenta, where *PLAC1* colocalized with chorionic gonadotropin beta (CGB) subunit transcripts (Fig. 5A,B). *PLAC1* transcripts were minimally expressed in cytotrophoblast (CTB) and extravillous (EVT) cell columns. These observations conflicted with an earlier report that provided evidence for PLAC1 expression in the EVT cell column (Chang et al., 2014). We next examined *PLAC1* expression profiles in single-nucleus RNA-seq datasets for human placenta tissue and trophoblast organoids (Keenen et al., 2025)*. PLAC1* expression was restricted to STB and not found in CTB or EVT cells (Fig. S12). Our PLAC1 *in situ* hybridization results and interrogation of single-nucleus RNA-seq datasets suggest that PLAC1 may not be a major contributor to EVT cell development and function.

The EVT cell column of the human placenta and the junctional zone of the rat placenta represent analogous structures and sources of invasive trophoblast cells in the respective species (Soares et al., 2018). Species differences in *PLAC1* expression within the placentation site suggest that PLAC1 may serve different roles in human versus rat placentation.

### PLAC1 contributions to human trophoblast cell differentiation

Human TSCs represent an effective model for elucidating regulatory mechanisms controlling trophoblast cell differentiation (Okae et al., 2018; Shimizu et al., 2023). Next, we evaluated the expression of PLAC1 in CT27 (X,X) human TSCs maintained in the stem state or differentiated into STB or EVT cell lineages. Differentiation of human TSCs is characterized by specific transcript profiles in each developmental state (Fig. S13A-C). PLAC1 was upregulated when human TSCs were differentiated into STB but not EVT cells (Fig. 5C,D), which is consistent with expression of *PLAC1* in STB of the first trimester human placenta (Fig. 5A, Fig. S12).

#### PLAC1 involvement in STB differentiation

A loss-of-function approach was used to investigate the role of PLAC1 in STB development. Control and *PLAC1* shRNAs were delivered to human TSCs in the stem state by lentiviral-mediated transduction. Disruption of PLAC1 transcript and protein expression was confirmed in cells expressing *PLAC1* shRNAs under STB conditions (Fig. 5E,F). Disruption of PLAC1 in STB affected cell morphology (Fig. S13D) and led to decreased cell fusion efficiency and secretion of chorionic gonadotropin (CG) (Fig. 5G,H). Transcriptomes of control shRNA- versus *PLAC1* shRNA-expressing TSCs after STB-3D differentiation were interrogated by RNA-seq. Depletion of PLAC1 resulted in 651 differentially regulated transcripts, including 264 downregulated transcripts and 387 upregulated transcripts (Fig. 5I, Table S4). Overall, there were minimal similarities between rat and human transcript profiles. KEGG and GSEA highlighted effects of PLAC1 on cell cycle regulation (Fig. S13E,F), which showed similarities with KEGG and GSEA analysis of RNA-seq datasets from PLAC1-deficient rat placentas and rat TSCs (Figs S5, S6 and S10). MKI67 expression and Bromodeoxyuridine (BrdU) incorporation were upregulated in PLAC1-depleted human TSCs after STB differentiation (Fig. S13G,H). Depletion of PLAC1 in the human TSC stem state did not significantly affect cell proliferation (Fig. S13H). PLAC1 was similarly shown to contribute to the differentiation of STB from CT29 (X,Y) human TSCs (Fig. S14, Table S5).

We also evaluated the role of PLAC1 in human TSC-derived trophoblast organoids (TOs), which are an effective model for investigating STB development (Karvas et al., 2022; Shannon et al., 2024; Yang et al., 2024). Efficient disruption of PLAC1 protein expression was confirmed in TOs expressing PLAC1-targeting shRNAs (Fig. 5J). Depletion of PLAC1 did not impair the ability of TSCs to form TOs (Fig. 5K); however, TO morphology was altered with the appearance of a thicker CTB layer, as indicated by an expanded presence of CDH1-positive cells (Fig. 5L) and reduction in CGB levels. Consistent with our findings in TSC monolayer cultures, PLAC1-depleted TOs exhibited a significant downregulation of STB-associated transcripts (*CSH1*, *SDC1*, *ERVFRD-1*, *ERVW-1* and *CGB*; Fig. 5M).

BeWo choriocarcinoma cells represent another model used extensively to investigate STB development (Orendi et al., 2010; Wice et al., 1990). BeWo choriocarcinoma cells express PLAC1; however, the impact of PLAC1 disruption in BeWo cells did not parallel our observations with human TSCs (Fig. S15, Table S6). These latter observations may reflect the actions of PLAC1 on the transformed character of BeWo cells (Fant et al., 2010).

#### PLAC1 involvement in EVT cell differentiation

Although PLAC1 expression is not a prominent feature of EVT cells (Fig. 5; Keenen et al., 2025), since an earlier report provided evidence for the involvement of PLAC1 in the regulation of EVT cell differentiation (Chang et al., 2014), we tested the impact of PLAC1 depletion on EVT cell differentiation using human TSC monolayer, spheroid and TO culture systems. The effects of PLAC1 depletion on EVT cell differentiation were modest. PLAC1 depletion did not impair the ability of TSCs to differentiate into EVT cells, as assessed by cell morphology and expression of *HLA-G*, *CCR1* and *NOTUM* (Fig. S16A,B). A modest decrease in cell migration was observed (Fig. S16C). In TOs, PLAC1 depletion did not impede the formation of EVT cells (Fig. S16D), but, in contrast, PLAC1 depletion did have a more-pronounced effect on the expression of EVT cell-associated transcripts (*HLA-G*, *CCR1* and *NOTUM*; Fig. S16E). This PLAC1 action in TOs likely reflects an indirect effect on EVT cell differentiation mediated by PLAC1 acting within STB, which may help explain the effects of PLAC1 disruption on EVT cell differentiation from placental explants (Chang et al., 2014).

In summary, PLAC1 contributes to the regulation of human STB development and function. We next sought to gain insight into the actions of PLAC1 within the trophoblast cell.

### PLAC1–FURIN interactions

PLAC1 has been reported to physically interact with FURIN, a member of the proprotein convertase family (Li et al., 2018; Lin et al., 2021; Shi et al., 2018). Notably, FURIN has been implicated in the regulation of human STB development (Morosin et al., 2021; Zhou et al., 2013), a cellular process linked to PLAC1 action (see Fig. 5). Consequently, we investigated PLAC1–FURIN interactions computationally and in trophoblast cell lineage development.

#### Computational analysis of PLAC1–FURIN interactions

**Fig. 6.**
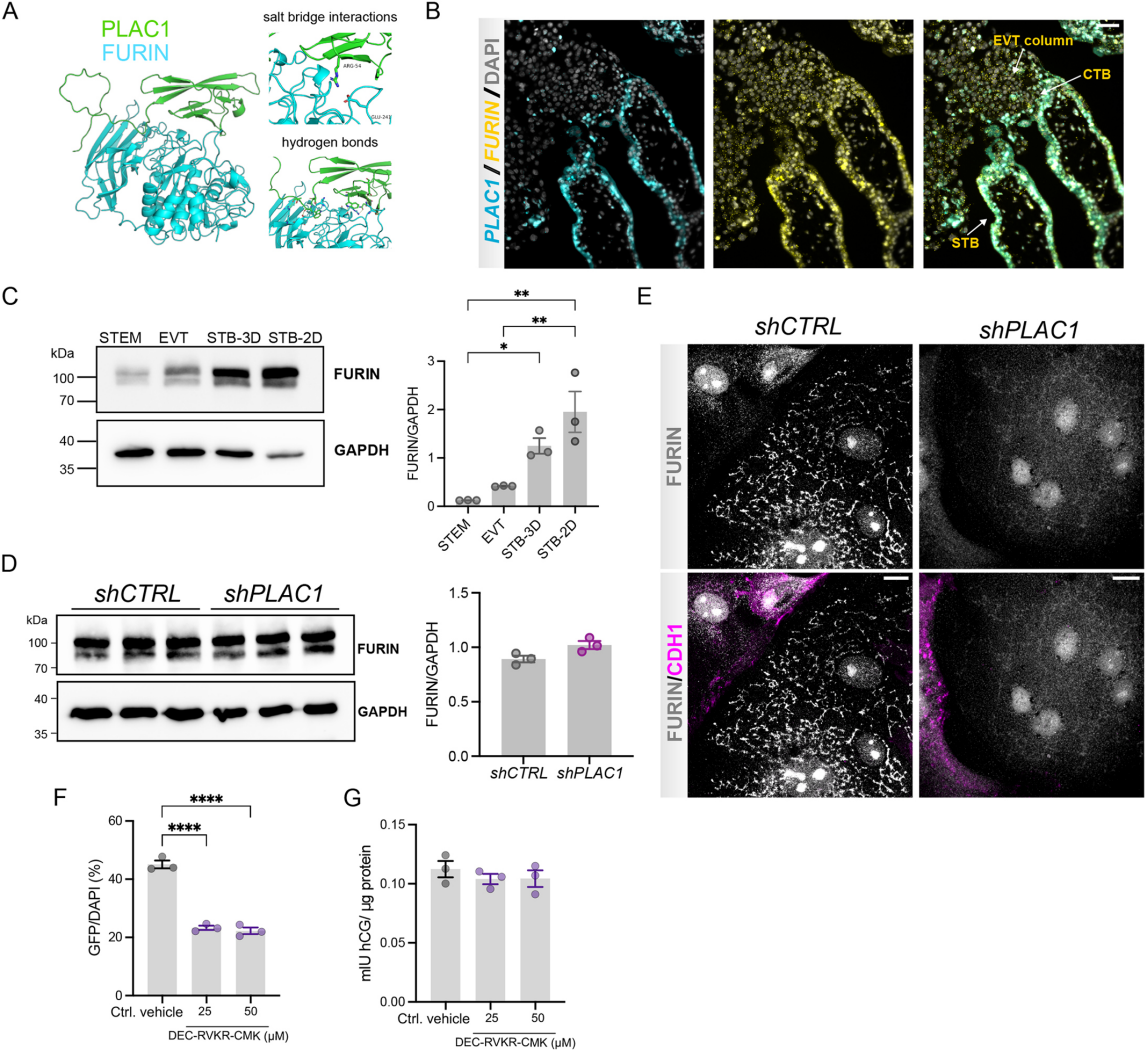
**Computational interactions of PLAC1-FURIN and involvement in trophoblast cell lineage development.** (A) Predicted model of human PLAC1 and FURIN interaction determined by AlphaFold 3. (B) Representative images of *PLAC1* (cyan) and *FURIN* (yellow) transcripts detected by *in situ* hybridization in tissue sections from the first trimester human placenta (11 weeks). Images are representative of 3 samples. (C) Western blotting analysis of FURIN in human TSCs in the stem cell state and following EVT cell and STB differentiation and quantification. FURIN protein was normalized to GAPDH (right). (D) Western blot analyses of FURIN in control shRNA (*shCTRL*)- and PLAC1 shRNA (*shPLAC1*)-treated human TSCs in the STB differentiation state. (E) Representative confocal images of FURIN detected by immunostaining in *shCTRL-* and *shPLAC1*-treated human TSCs in the STB differentiation state. CDH1 expression is observed in unfused cells. (F) Cell fusion assessment of treated human TSCs in the STB differentiation state with control vehicle or FURIN inhibitor (25 and 50 μM, DEC-RVKR-CMK). (G) CG enzyme-linked immunoassay measurements of human TSCs in the STB differentiated state treated with vehicle or FURIN inhibitor (25 and 50 μM, DEC-RVKR-CMK). Data are presented as mean±s.e.m. Each dot represents a biological replicate (*n*=3). *P<0.05, **P<0.005, ****P<0.0001 (for C,F,G, one-way analysis of variance and Tukey's multiple comparisons test; for D, unpaired *t*-test). CTB, cytotrophoblast; EVT, extravillous trophoblast; STB, syncytiotrophoblast*.* Scale bars: 50 μm (A); 20 μm (E).

AlphaFold 3 was used to determine whether there was a computational framework for PLAC1–FURIN interactions (Abramson et al., 2024). Five independent models of PLAC1–FURIN interaction were generated. A consensus was established for similar binding sites between both human and rat PLAC1 with human and rat FURIN proteins, respectively (Fig. 6A, Fig. S17). The N-terminal domain of PLAC1 was modeled to sit on one of the surfaces of the peptidase domain flanking the substrate-binding groove of FURIN, making several interactions with the loop regions of the peptidase domain (Fig. 6A). Interestingly, the disordered region following the N-terminal domain of PLAC1 shares a Ser-Pro-Trp-Leu-Thr-Lys-Pro sequence between rat and human, and that is modeled to interact with a region on the P/Homo B domain adjacent to the substrate-binding site of FURIN. In these models, Trp-101 fits into a hydrophobic cleft, Leu-102 makes hydrophobic contacts and Pro-105 rests on an arginine. Based on this model, it could be hypothesized that PLAC1 physically interacts with FURIN.

#### PLAC1 and FURIN expression within rat and human placentation sites

FURIN transcripts were localized to the same placental compartments expressing PLAC1 transcripts (Fig. S18, Fig. 6B). In the rat, *Furin* was localized throughout the placenta, and colocalized with *Plac1* in the junctional zone as well as in interstitial invasive trophoblast cells located within the uterine–placental interface (Fig. S18A). In the human, *FURIN* transcripts were also widespread and colocalized with *PLAC1* transcripts in STB (Fig. 6B). FURIN expression was consistent with PLAC1 expression in differentiating rat TSCs (Fig. S18B) and human TSC-derived-STB cells (Fig. 6C). Thus, based on computational modeling and tissue distribution, a case can be constructed for potential PLAC1 and FURIN interactions.

#### PLAC1 impacts intracellular distribution of FURIN in TSCs

Loss of PLAC1 (shPLAC1-treated cells) did not affect the total amount of FURIN (Fig. 6D, Fig. S19A) or the enzymatic activity of FURIN against a known FURIN substrate in human TSC-derived STB cells or differentiated rat TSCs (Fig. S19B); however, loss of PLAC1 did affect the intracellular distribution of FURIN in both models (Fig. 6E, Fig. S19C). In the presence of PLAC1, FURIN was present in both the nucleus and mesh-like structures within the cytoplasm (Fig. 6E). In contrast, in the absence of PLAC1, FURIN was restricted to the nucleus and the perinuclear cytoplasm, while the mesh-like immunoreactive pattern was absent (Fig. 6E). These observations indicate that PLAC1 could be modulating the intracellular distribution of FURIN (Anderson et al., 1997).

#### FURIN actions on human STB

A small molecule FURIN inhibitor (DEC-RVKR-CMK) was used to assess a role for FURIN in STB development. Disruption of FURIN activity interfered with human TSC differentiation into STB as assessed by cell fusion (Fig. 6F), but did not significantly affect CG secretion (Fig. 6G), nor did FURIN disruption affect *MKI67* expression (Fig. S20). Thus, PLAC1 (Fig. 5G) and FURIN (Fig. 6F) possess overlapping actions on STB development.

In summary, PLAC1 is a conserved regulator of rat and human placentation; however, the specific subpopulation of trophoblast cells primarily impacted by PLAC1 is species specific. The actions of PLAC1 are linked, at least in part, to its interactions with FURIN.

## DISCUSSION

In this study, we have examined the biology of the X-linked gene *Plac1* in pregnancy and placentation. PLAC1 plays a fundamental role in the regulation of hemochorial placentation but acts on different trophoblast cell lineages in a species-specific manner. We show that in the rat, maternally inherited PLAC1 restrains development of trophoblast cells within the junctional zone compartment of the placenta, yet promotes cell-autonomous development of invasive trophoblast cells infiltrating the uterine parenchyma. These opposing actions were unexpected but may be informative in understanding maternal and paternal genetic conflicts in pregnancy. In contrast, PLAC1 does not possess marked actions on the invasive trophoblast cell lineage in the human placenta but instead acts to ensure appropriate development of the STB lineage, a crucial component of the endocrine and barrier functions of the placenta. The actions of PLAC1 on trophoblast cell differentiation are mediated, at least in part, through its interactions with FURIN.

The PLAC1-deficient rat placenta is characterized by a dramatically expanded junctional zone and a minimally affected labyrinth zone. The junctional zone is situated at the interface with the uterus, principally targets maternal tissues through its production of hormones, and seeds the uterus with invasive trophoblast cells (John, 2013; Soares et al., 2012, 1996), while the labyrinth zone is essential for maternal–fetal nutrient transfer (Dilworth and Sibley, 2013; Knipp et al., 1999). Delivery of nutrients is rate limiting for fetal development (Desforges and Sibley, 2010). Thus, it is understandable that the functionality of the large PLAC1-deficient placenta, which is biased towards junctional zone growth, did not enhance nutrient delivery and fetal size. John and colleagues have attributed the size of the junctional zone and its capacity for hormone production to modulation of the maternal brain and the acquisition of maternal care behaviors that favor offspring survival (Creeth et al., 2018; John, 2019). Junctional zone ligands responsible for these actions on the maternal brain have not been elucidated. Prolactin and some of its paralogs expressed in the placenta engage the prolactin receptor, act on the maternal brain, and promote the development of maternal behaviors (Bridges and Grattan, 2019; Shingo et al., 2003; Soares et al., 2007). Some members of the prolactin family exhibited diminished expression in the PLAC1-deficient junctional zone (present study). How the increase in junctional zone mass in the PLAC1-deficient placenta affected circulating ligands targeted to maternal tissues, and especially the brain and substrates affecting maternal care behavior, remains to be determined.

There are intriguing temporal and spatial aspects to the actions of PLAC1 in the rat placenta. Presumably, PLAC1 modulation of junctional zone development is established during the early phases of placentation when it is at its peak expression. To extend this logic, during the early stages of placentation PLAC1 contributes to cell fate decisions impacting the constituents of the junctional zone, including trophoblast giant cells, spongiotrophoblast cells, glycogen cells, invasive trophoblast progenitor cells, and maybe others (Simmons and Cross, 2005; Soares et al., 2012, 1996). The junctional zone arises, at least in part, through PLAC1 favoring development of some trophoblast cell lineages and restraining the expansion of other lineages. PLAC1 also establishes a smooth interface between the junctional and labyrinth zones. The smooth interface facilitates separation of the rat placental compartments by simple mechanical dissection (Ain et al., 2006). In the absence of PLAC1, the junctional zone and labyrinth zone border becomes interdigitated, and the zones are difficult to separate. The mixing of placental zones is extreme in some experimental manipulations, such as X-linked placental dysplasia observed in interspecies mouse hybrids (Hemberger et al., 1999; Zechner et al., 1996) and placentomegaly observed in nuclear transfer-mediated cloning of mice (Tanaka et al., 2001). Dysregulated PLAC1 is a potential contributor to the observed anomalies in placentas from both interspecies hybrid and cloning manipulations (Singh et al., 2004; Suemizu et al., 2003). Precise molecular mechanisms underlying the actions of PLAC1 on junctional zone cellular dynamics and establishment of the integrity of the intraplacental compartments are unknown.

The junctional zone of the rat placenta serves as a niche for invasive trophoblast progenitor cell development (Soares et al., 2018, 2012). In the rat, PLAC1 propels invasive trophoblast cell development, especially the expansion of interstitial invasive trophoblast cells that infiltrate the uterine stroma situated between uterine spiral arteries, with lesser effects on the endovascular invasive trophoblast cell population. This observation indicates that regulatory mechanisms controlling interstitial and endovascular invasive trophoblast cells are distinct. Evidence also supports a cell-autonomous role for PLAC1 in interstitial invasive trophoblast cell development. A diminution in interstitial invasive trophoblast cells is either not relevant for fetal development or is compensated for by other unknown maternal and/or placental adaptations. NK cells at the uterine–placental interface exhibit a reciprocal relationship with invasive trophoblast cells (Ain et al., 2003). NK cells are abundant within the midgestation uterine–placental interface. As invasive trophoblast cells enter the uterine–placental interface, NK cells are expelled and relocated towards the periphery of the placentation site. Failure of interstitial invasive trophoblast cell invasion into the uterus is associated with a uterine–placental interface infiltrated with NK cells (Dominguez et al., 2025; and present study). The retention of NK cells in the late-gestation uterine–placental interface may be due to the absence of interstitial invasive trophoblast cell signals instructing NK cells to vacate. NK cells act to remodel uterine spiral arteries during the establishment of pregnancy (Chakraborty et al., 2011; Zhang et al., 2011) and may contribute to successful pregnancy outcomes in the absence of intrauterine invasive trophoblast cells.

The EVT cell column of the human placenta is a structure analogous to the junctional zone of the rat placenta and similarly a site where the invasive trophoblast cell lineage arises (Knöfler et al., 2019; Pollheimer et al., 2018; Soares et al., 2018). An earlier report suggested that PLAC1 regulates aspects of EVT cell development in human placentation (Chang et al., 2014). PLAC1 expression was observed in first and second trimester EVT cells and silencing PLAC1 inhibited trophoblast cell migration from first trimester EVT explants (Chang et al., 2014). In contrast, in our analyses PLAC1 expression was low in EVT cell columns of the first trimester human placentation site and in human TSCs induced to differentiate into EVT cells. We did not observe a significant role for PLAC1 in EVT cell differentiation; instead, we detected prominent PLAC1 expression in the STB compartment of the first trimester human placentation site and demonstrated a role for PLAC1 in human STB development. These latter observations are consistent with the reported distribution of *PLAC1* in the human placentation site (Arutyunyan et al., 2023; Fant et al., 2007, 2002; Keenen et al., 2025; Vento-Tormo et al., 2018) and the actions of PLAC1 on STB (Chang et al., 2016).

The mechanism of action of PLAC1 on cell function has been a mystery. Some insights were gained from PLAC1 immunoprecipitation-mass spectrometry experiments in oocytes and cancer cells. PLAC1 was shown to associate with several proteins, including FURIN (Li et al., 2018; Lin et al., 2021; Shi et al., 2018). FURIN is a protease with a wide range of substrates and effectively converts a precursor protein to a mature biologically active protein (Seidah and Prat, 2012; Thomas, 2002). In trophoblast cells, FURIN has been implicated in regulating syncytialization (Zhou et al., 2013). Among FURIN substrates in trophoblast cells are syncytins (Chen et al., 2008), which promote the fusion of CTBs to STB (Blaise et al., 2003; Chen et al., 2008; Dupressoir et al., 2009; Roberts et al., 2021). Through computational analyses, we identified potential physical interactions between PLAC1 and FURIN proteins. Experimentally, PLAC1 and FURIN mRNAs were colocalized within trophoblast cells of the developing placenta. Most importantly, silencing PLAC1 altered the intracellular localization of FURIN. These observations support a role for PLAC1 affecting trophoblast cells in the developing placenta, at least in part through modulation of FURIN.

Collectively, the findings presented here are consistent with PLAC1 possessing a regulatory role in placentation and highlight a striking species difference in the biology of PLAC1 in hemochorial placentation. In the rat, PLAC1 exhibits a fundamental role in development of trophoblast cell lineages situated at the uterine–placental interface, whereas in the human PLAC1 contributes to establishment of trophoblast cells within the placental–fetal barrier. Central to differences in the biology of PLAC1 is its expression in nonhomologous trophoblast cell lineages (rat invasive trophoblast cells versus human STB). Thus, it is evident that regulatory mechanisms controlling cell-specific expression of PLAC1 differ between the rat and human. These differences could be intrinsic to rat versus human PLAC1 genes and/or in the cellular machinery controlling PLAC1 expression in the rat versus human. PLAC1 is conserved across mammalian species (Devor, 2014); however, limited knowledge is available on PLAC1 in mammals other than the mouse, rat and human. Comparative experimentation into the biology of PLAC1 should lead to valuable insights into the evolution of the placenta. Furthermore, this alternative purposing of the same gene for different functions within the placenta across a range of mammalian species may be a common phenomenon (Stadtmauer et al., 2025).

A conflict or competition of maternal versus paternal genomes has been asserted as an important theme underlying placental development (Baker, 2024; Haig, 1993; Moore and Haig, 1991). In its simplest form, as related to the placenta, the ‘parental conflict theory’ is predicated on the activities of paternal alleles driving placental growth and maximizing maternal nutrient delivery to the fetus, whereas maternal alleles restrain placental growth and the re-direction of maternal nutrients to the fetus (Fowden et al., 2011; Moore, 2012). A balance is achieved that protects maternal, paternal and offspring interests. The biology of imprinted and X-linked genes has provided core evidence supporting the parental conflict theory (Fowden et al., 2011; Moore, 2012; Moore and Haig, 1991). There appear to be exceptions to the theory where apparent roles are reversed (Cleaton et al., 2014; Fowden et al., 2011; Iwasa, 1998; Moore, 2012). PLAC1 presents a mixed message, especially regarding its involvement in rat placentation. At one level, the parental conflict theory is supported in that maternally inherited PLAC1 restrains growth of the major source of placental hormones targeted to the mother. Seemingly, these hormones are driving maternal adaptations that promote fetal growth. However, this supposition is a hypothesis because we know little about the actions of placental hormones in the rat or mouse (Soares et al., 2018, 2007). Another important role for PLAC1 has emerged, which at face value seems contradictory to the parental conflict theory. In the rat, PLAC1 drives invasive trophoblast cell development and is essential for the invasion of trophoblast cells into the uterus. This maternal intrusion is driven by a maternally inherited allele. Alternatively, this apparent contradiction may be overly simplistic and what makes trophoblast cell incursion into the uterus compatible with the mother, and the parental conflict theory, is that maternal alleles are controlling the invading trophoblast cells.

### Study limitations

A limitation of this study is the lack of a reliable antibody suitable for PLAC1 immunolocalization and immunoprecipitation. Despite testing multiple commercially available antibodies, we could not identify an antibody that provided specific immunostaining or that was effective for PLAC1 immunoprecipitation. Consequently, we were unable to directly assess the spatial relationship or potential interactions between PLAC1 and FURIN at the protein level.

## MATERIALS AND METHODS

### Animals and tissue collection

Holtzman Sprague-Dawley rats were maintained on 14:10 h (light:dark cycle), with free access to food and water. Timed pregnancies were established by co-housing male (>10 weeks of age) and female (8-12 weeks of age) rats. Presence of sperm in the vaginal lavage was an indicator of mating and considered as gd 0.5. Pseudopregnancy was established by mating with vasectomized male rats. Pregnant rats were euthanized on gd 9.5, 11.5, 13.5, 15.5, 18.5 or 20.5. Body weights were recorded. Some placentation sites were frozen in dry ice-cooled heptane and stored at −80°C until used for histological analyses, whereas other placentation sites were dissected, placentas and fetuses weighed, and tissues frozen in liquid nitrogen and stored at −80°C until used for RNA or protein analyses (Ain et al., 2006). The genotype and genetic sex of each embryo associated with a placentation site was determined by PCR using previously described procedures (Dhakal and Soares, 2017). The University of Kansas Medical Center (KUMC) Animal Care and Use Committee approved all protocols used in this study.

### Human placenta tissues

Paraffin-embedded, de-identified, first trimester human placenta tissue sections were obtained from the Lunenfeld-Tanenbaum Research Institute (Mount Sinai Hospital, Toronto, Canada). Placenta specimen collections were performed following written informed consent. Institutional approval was obtained from Human Research Ethics Review Committees at the University of Toronto and the KUMC.

### Generation of a mutant *Plac1* rat model

The *Plac1* mutant rat model was generated using CRISPR/Cas9 genome editing with guide RNAs (gRNAs) targeting exon 3 of the rat *Plac1* gene (NM_001024894.1; Table S7) using previously described methods (Iqbal et al., 2021; Kozai et al., 2023; Muto et al., 2021). *Plac1*-targeted gRNAs were generated using duplex CRISPR RNA and trans-activating CRISPR RNA (Integrated DNA Technologies). Embryonic day 0.5 zygotes were harvested from superovulated females on the morning following mating with males of proven fertility (Shao et al., 2014). Zygotes were electroporated with a mixture of gRNAs for *Plac1* (35 ng/ml) and Cas9 protein (1 ng/ml) prepared in Tris-EDTA buffer (pH 7.4). The NEPA 21 electroporator (NEPA GENE) was used to transfer the gene-editing reagents (Kaneko, 2017). Manipulated zygotes were transferred to oviducts of pseudopregnant rats (20-30 zygotes per rat). Offspring were screened for *Plac1* mutations by PCR and verified by DNA sequencing. A founder rat with a 469 bp deletion within exon 3 was confirmed. The founder was subsequently backcrossed with wild-type rats to confirm germline transmission and to establish a colony. Primers used for genotyping are listed in Table S8.

### Rat TSC culture

TSCs originally derived from rat blastocysts (Asanoma et al., 2011) were cultured in rat TSC medium [RPMI 1640, 20% (vol/vol) fetal bovine serum (FBS, Thermo Fisher Scientific), 100 μm 2-mercaptoethanol (M7522, Sigma-Aldrich), 1 mM sodium pyruvate (11360-070, Thermo Fisher Scientific), 50 μM penicillin (15140122, Thermo Fisher Scientific) and 50 U/ml streptomycin (15140122, Thermo Fisher Scientific)] supplemented with 70% rat embryonic fibroblast-conditioned medium prepared as previously described (Asanoma et al., 2011), fibroblast growth factor 4 (FGF4, 25 ng/ml; 100-31, Peprotech) and heparin (1 μg/ml; H3149, Sigma-Aldrich). TSCs were induced to differentiate by culturing for 15 days in rat TSC medium with 1% FBS and without FGF4, heparin and rat embryonic fibroblast-conditioned medium.

### Human TSC culture

Human TSCs (CT27, X,X; CT29, X,Y) were maintained in the stem state as previously described (Okae et al., 2018). Cells were cultured in dishes pre-coated with type IV collagen (5 μg/ml; C5557, Sigma-Aldrich). TSCs were maintained in Complete TS Cell Medium [DMEM/F12 (11320033, Thermo Fisher Scientific), 100 μm 2-mercaptoethanol, 0.2% (vol/vol) FBS, 50 μM penicillin, 50 U/ml streptomycin, 0.3% bovine serum albumin (BSA; BP9704100, Thermo Fisher Scientific), 1% insulin-transferrin-selenium-ethanolamine solution (ITS-X; vol/vol, 51500056, Thermo Fisher Scientific), 1.5 μg/ml L-ascorbic acid (A8960, Sigma-Aldrich), 50 ng/ml epidermal growth factor (EGF; E9644, Sigma-Aldrich), 2 μM CHIR99021 (04-0004, Reprocell), 0.5 μM A83-01 (04-0014, Reprocell), 1 μM SB431542 (04-0010, Reprocell), 0.8 mM valproic acid (P4543, Sigma-Aldrich) and 5 μM Y27632 (04-0012-02, Reprocell)].

#### EVT cell differentiation

EVT cell differentiation was performed as previously described (Okae et al., 2018). Human TSCs were plated into 6-well plates precoated with 1 μg/ml type IV collagen and cultured in EVT Cell Differentiation Medium: DMEM/F12 supplemented with 100 μM 2-mercaptoethanol, 50 U/ml penicillin and 50 μg/ml streptomycin, 0.3% BSA, 1% ITS-X, 100 ng/ml neuregulin 1 (NRG1; 5218SC, Cell Signaling Technology), 7.5 μM A83-01, 2.5 μM Y27632, 4% KnockOut Serum Replacement (KSR; 10828028, Thermo Fisher Scientific) and 2% Matrigel (CB-40234, Thermo Fisher Scientific). On day 3 of EVT cell differentiation, the medium was replaced with the EVT Cell Differentiation Medium without NRG1, and the Matrigel concentration was decreased to 0.5%. On day 6 of EVT cell differentiation, the medium was replaced with EVT Cell Differentiation Medium without NRG1 or KSR and with Matrigel at a concentration of 0.5%. Cells were analyzed on day 8 of EVT cell differentiation.

#### STB differentiation

STB differentiation was performed as previously described (Okae et al., 2018). Two protocols were used to promote STB differentiation: two-dimensional (STB-2D) and three-dimensional (STB-3D). In the STB-2D protocol, TSCs were plated in a 6-well plate pre-coated with 2.5 μg/ml type IV collagen at a density of 100,000 cells per well and cultured in STB-2D Medium [DMEM/F12, 50 U/ml penicillin, 50 μg/ml streptomycin, 0.15% BSA, 1% ITS-X solution (vol/vol), 200 μM L-ascorbic acid, 5% KSR, 2.5 μM Y27632, 2 μM forskolin (F6886, Sigma-Aldrich)]. The medium was replaced on day 3, and the cells were analyzed on day 6 of STB cell differentiation. In the STB-3D protocol, TSCs were plated in 6 cm Petri dishes at a density of 300,000 cells per dish without type IV collagen coating and cultured in STB-3D Medium [DMEM/F12, 50 μM penicillin, 50 U/ml streptomycin, 0.15% BSA, 1% ITS-X solution (vol/vol), 5% KSR, 2.5 μM Y27632, 2 μM forskolin and 50 ng/ml EGF]. The cells aggregated and grew as 3D structures. On day 3 of cell differentiation, 3 ml of fresh STB-3D medium was added to the culture dishes. Cells were analyzed on day 6 of STB differentiation.

#### TOs

Human TSCs were used to establish TOs as previously described (Karvas et al., 2022) with minor modifications. Briefly, 3000 TSCs were embedded in 30 μl Matrigel (75% in Advanced DMEM/F12) and plated as droplets in 24-well plates. After 30 min of polymerization at 37°C, 500 μl of modified TO medium (Shannon et al., 2024) [mTOM: Advanced DMEM/F12 (12-634-010, Thermo Fisher Scientific) supplemented with 1× N2 (17502-048, Thermo Fisher Scientific), 1× B27 (17504044, Thermo Fisher Scientific), 100 μg/ml Primocin (ant-pm-1, InvivoGen), 1.25 mM N-acetyl-L-cysteine (A9165, Sigma-Aldrich), 2 mM L-glutamine (25030081, Gibco), 1 μM A83-01, 3 μM CHIR99021, 2 μM Y-27632, 100 ng/ml EGF, 100 ng/ml R-spondin 1 (4645-RS, Bio-Techne)] was added. Culture medium was replaced every other day, and TOs were passaged every 8-10 days. For passaging, Matrigel droplets were digested with TrypLE for 20 min at 37°C, followed by three washes with Advanced DMEM/F12. Large aggregates were removed using a 40 μm cell strainer, and 3000-5000 cells were reseeded per 30 μl Matrigel droplet. For EVT cell differentiation, dissociated TO cells were embedded in 30 μl Matrigel droplets in 24-well plates and maintained in mTOM for 3 days. The culture medium was then replaced with EVT Cell Differentiation Medium for 5 days with daily medium changes (500 μl per well). EVT cells were analyzed following an additional 3 days of culture in EVT differentiation medium lacking NRG1.

Experimentation with human TSCs was approved by the KUMC Human Research Protection Program and the KUMC Human Stem Cell Research Oversite committee.

### BeWo cell culture

Choriocarcinoma-derived BeWo cells (Pattillo and Gey, 1968) were obtained from the American Type Culture Collection. BeWo cells were maintained in DMEM/F12 supplemented with 10% FBS, 100 U/ml penicillin and 100 μM streptomycin. Syncytialization was induced in BeWo cells by treatment with 20 μM forskolin (Msheik et al., 2019; Orendi et al., 2010).

### Histological and immunohistochemical analyses

All histological analyses of rat placentation sites were performed on 8 μm tissue sections embedded in Optimal Cutting Temperature (OCT) compound (88191, PANTek Technologies). Sections were fixed in 4% paraformaldehyde (PFA) for 10 min at room temperature and permeabilizated with 100% methanol for 15 min at room temperature. Tissue sections were blocked with 10% goat serum (50062Z, ThermoFisher Scientific) for 1 h prior to incubation with primary antibodies (diluted in blocking solution) overnight at 4°C. Cytokeratin immunostaining was used to visualize invasive trophoblast cells within the uterine–placental interface (Ain et al., 2003; Konno et al., 2007; Rosario et al., 2008) and vimentin immunostaining was used to discriminate between junctional and labyrinth zone compartments of the rat placenta (Konno et al., 2007). We localized cytokeratin and vimentin using a mouse monoclonal antibody to pan-cytokeratin conjugated to fluorescein isothiocyanate (1:300; F3418, Sigma-Aldrich) and a mouse monoclonal antibody to vimentin (1:300; sc-6260, Santa Cruz Biotechnology), respectively. MCT1 and MCT4 antibodies were used to assess labyrinth zone integrity (1;300; AB1286-I and AB3314P, Sigma-Aldrich). Ki-67 antibody was used to assess proliferative cells in the junctional zone (1:300; ab16667, Abcam). Goat anti-mouse immunoglobulin conjugated to Alexa Fluor 488 (1:300; A11031, Thermo Fisher Scientific), goat anti-rabbit immunoglobulin conjugated to Alexa Fluor 568 (1:300; A11011, Thermo Fisher Scientific) and donkey anti-chicken immunoglobulin conjugated to Alexa Fluor 568 (1:300; A78950, Thermo Fisher Scientific) were used as secondary antibodies for immunofluorescence, diluted in blocking solution and incubated for 1 h at room temperature. Nuclei were visualized using 4′6-diamidino-2-phenylindole (DAPI) (00-4959-52, Thermo Fisher Scientific). Slides were stored at 4°C until microscopic inspection. Processed tissue sections were examined, and images captured with a Nikon 90i upright microscopes with Photometrics CoolSNAP-ES monochrome cameras (Roper) and 2047 Zeiss Axio Observer 7 with Apotome III and AI Sample Finder. Image processing and morphological measurements were determined with NIH ImageJ software as previously described (Konno et al., 2007; Rosario et al., 2008).

### Western blotting

Tissue and cell lysates were collected in radioimmunoprecipitation assay lysis buffer system, which includes a protease inhibitor cocktail consisting of phenylmethylsulfonyl fluoride and sodium orthovanadate (sc-24948A, Santa Cruz Biotechnology). Protein concentrations were determined using the detergent compatible protein assay (5000112JA, Bio-Rad). Proteins were separated on sodium dodecyl sulfate-polyacrylamide gels (50 μg/lane) and transferred to polyvinylidene difluoride membranes (10600023, GE Healthcare) for 1 h on ice. Membranes were blocked in 5% non-fat milk in Tris-buffered saline (TBS, pH 7.4)-Tween 20 (0.1%) for 1 h at room temperature. Antibodies to PLAC1 (human: 1:100, sc-365919, Santa Cruz Biotechnology; rat: 1:500, MBS460437, MyBioSource), FURIN (human: 1:500, ab3467, Abcam; rat: 1:50, sc-133141, Santa Cruz Biotechnology) and GAPDH (1:10,000, AM4300, Thermo Fisher Scientific) were diluted in 5% non-fat milk supplemented TBS-Tween 20 (0.1%) and incubated at 4°C overnight. Immunoreactive proteins were detected with secondary antibodies consisting of horseradish peroxidase (HRP)-conjugated goat anti-rabbit IgG (1:5000l 7074P2, Cell Signaling Technology) or HRP-conjugated goat anti-mouse IgG (1:5000; 7076S, Cell Signaling Technology) and the Luminata™ Crescendo Western HRP substrate (EMD Millipore).

### *In situ* hybridization

Detection of transcripts was performed on fixed, frozen rat placentation sites and on paraffin-embedded human placenta tissue sections using the RNAscope Multiplex Fluorescence Reagent Kit v2 (Advanced Cell Diagnostics), according to the manufacturer's instructions. Probes were prepared by Advanced Cell Diagnostics to detect rat *Plac1* (860141; NM_001024894.1; target region: 3-944), rat *Prl7b1* (860181-C2; NM_153738.1; target region: 28-900), rat *Prf1* (871601-C2; NM_017330.2; target region: 451-1452), rat *Furin* (1194731-C3, NM_019331.2; target region: 1786-2710), rat *Gcm1* (878381-C2, NM_017186.2; target region: 533-1485), rat *Pcdh12* (1272141, NM_053944.1; target region: 1738-2615), rat *Psg29* (1269681, NM_001025641.1; target region: 446-2069), rat *Prl7a3* (1572721-C1,NM_022530.2; target region: 2-1014), human *PLAC1* (485801, NM_021796.3; target region: 2-1109), human *CDH1* (311091, NM_004360.3, target region: 263-1255), human *CGB* (454831-C2, NM_000737.3, target region: 3-425) and human *FURIN* (858621-C2, NM_002569.4; target region: 866-2121). Nuclei were visualized using DAPI. Fluorescence images were viewed and captured as described above.

### Migration assay

Trophoblast cell migration was assessed using a previously published method (Shimizu et al., 2023) with minor modifications. Briefly, 300,000 human TSCs maintained in the stem state were resuspended in a mixture of Matrigel (75%) and EVT Cell Differentiation Medium (25%). From this suspension, 1 μl droplets were transferred to 6-well plates to generate individual Matrigel domes. Domes were cultured in complete EVT Cell Differentiation Medium supplemented with 0.5% Matrigel at 37°C in a humidified atmosphere containing 5% CO₂. Culture medium was replaced on day 3, and cells were maintained for a total of 6 days. Migrating EVT cells were identified by immunocytochemistry using a phycoerythrin (PE)-conjugated anti-HLA-G antibody (1:300; ab24384, Abcam). Following a 15 min incubation at 37°C, samples were fixed with 4% PFA, and nuclei were counterstained with DAPI. Samples were imaged using a Zeiss Axio Observer 7 equipped with Apotome III and AI Sample Finder. Images were processed using NIH ImageJ, and the total area occupied by HLA-G^+^ cells was quantified and normalized to control samples.

### RNA-seq

RNA-seq analyses were performed on gd 13.5 placental and gd 18.5 junctional zone tissues from wild-type and *Plac1* mutant rats, and in rat TSCs, human TSCs and BeWo cells treated with control or PLAC1 shRNAs. Total RNA was isolated using the TRIzol reagent according to the manufacturer's instructions (Thermo Fisher Scientific) and used as a template for cDNA isolation with the High-Capacity cDNA Reverse Transcription kit (Applied Biosystems). cDNA libraries were prepared and sequenced using a NovaSeq 6000 by the KUMC Genomics Core.

### RNA-seq data analyses

For comprehensive details on the analysis methods employed, including specific options and parameters used, please refer to the GitHub repository: https://github.com/Tuteja-Lab/SoaresLab_PLAC1_bulkRNAseq.

#### Initial data processing

Raw data obtained from the sequencing facility were converted to fastq format using bcl2fastq (v2.20.0.422) to facilitate downstream analysis. FastQC (v0.11.9) was employed to assess the quality of the converted fastq files. Upon ensuring satisfactory quality, samples run on multiple lanes were merged.

The datasets generated from rat tissues and cells (‘PLAC1_placenta_13.5’, ‘PLAC1_JZ_18.5’ and ‘rTS_Diff_PLAC1_KD’), used the rat genome and annotation (version mRatBN7.2) (de Jong et al., 2022) downloaded from Ensembl (release 105; 85), while the data generated from human cells (‘hTS_CT27_ST3D_PLAC1_KD’, ‘CT29_ST3D_PLAC1_KD’ and ‘BeWo_PLAC1_KD’) used the human genome and annotation (version GRCh38.p13; 86) downloaded from GENCODE (Frankish et al., 2021) for analyses.

The genomes were first indexed using the STAR (v2.7.10b) (Dobin et al., 2013) genomeGenerate subcommand with annotations (in GTF format) to provide splice junction information while mapping. Read mapping was performed by using the STAR alignReads subcommand and was optimized for short reads with the options --outFilterScoreMinOverLread 0.3 and --outFilterMatchNminOverLread 0.3. Individual bam files for each sample file were used with featureCounts from the subread package to generate a combined count file. The count file was filtered to retain only protein-coding genes. The quality-control reports from all the pre-processing steps were aggregated using MultiQC (v1.17; 89).

#### Differential gene expression analysis

The raw count files were imported into R (v4.2.2) and analyzed to obtain differentially regulated transcripts using DESeq2 (v1.38.3) (Love et al., 2014). Specifically, transcripts with counts greater than ten across all conditions were retained and tested for differential expression with the Wald statistical test. The resulting *P*-values were corrected for multiple testing with Benjamini–Hochberg to control the false discovery rate. Differentially regulated transcripts were identified after filtering them according to a *P*-value (adjusted) ≤0.05 and absolute fold change ≥1.5.

#### Enrichment analysis

We performed GSEA (Subramanian et al., 2005) using the clusterProfiler (v4.6.2) (Wu et al., 2021) package in R. For rat genesets, org.Rn.eg.db (v3.16.0, Bioconductor) was used and for human differentially expressed genesets, orgDB was set to org.Hs.eg.db (v3.16.0; Bioconductor). Enrichment analysis was performed using the pre-ranked mode, where DEGs were ranked based on their log-fold changes and statistical significance obtained from DESeq2 analysis. These differentially expressed genesets were tested against the Molecular Signatures Database (MSigDB; v2023.2) (Liberzon et al., 2015), with ontology set to ‘ALL’, and minimum and maximum number of genes in the geneset set to 3 and 800, respectively. The *P*-value cutoff was set to 0.05 and the method for adjustment of multiple hypothesis testing was set to ‘BH’. The plots were generated using the built-in functions of clusterProfiler. Using similar settings, KEGG (Kanehisa and Goto, 2000) pathway enrichment was also performed using the clusterProfiler gseKEGG function.

#### Comparison of human RNA-seq data

After importing the normalized counts for each human placental cell experiment, (‘hTS_CT27_ST3D_PLAC1_KD’, ‘CT29_sST3D_PLAC1_KD’ and ‘BeWo_PLAC1_KD’) from DESeq2, we identified the top 500 variable genes using the rowVars function from matrixStats (v1.2.0). Subsequently, correlation plots depicting the log2 fold change of these genes were generated for each pairwise comparison using ggcorrplot (v0.1.4.999). Additional combined analyses of all human placental cells (‘hTS_CT27_ST3D_PLAC1_KD’, ‘CT29_sST3D_PLAC1_KD’ and ‘BeWo_PLAC1_KD’) were conducted after batch correcting the merged counts using ComBat-seq (v3.46.0) (Zhang et al., 2020). DESeq2 was used to convert the batch corrected counts to variance stabilizing transferred counts. Using the ‘dist’ function of DESeq2, sample-to-sample distances were calculated from the vst counts. The resulting distance matrix was plotted using the heatmap (v1.0.12) package in R.

### Differential gene expression cluster enrichment analysis

To assess the cellular context of DEGs in rat placental tissues and trophoblast cells, we integrated bulk differential gene expression datasets with a published mouse placental single-nucleus RNA-seq cluster annotation (Marsh and Blelloch, 2020). Clusters corresponding to syncytiotrophoblast and trophoblast giant cells were excluded to focus the analysis on junctional zone-associated trophoblast cell populations. DEGs were obtained from independent RNA-seq analyses at gd 13.5 and 18.5 placental tissues and from rat TSCs (see above). Genes were classified as upregulated or downregulated based on adjusted *P*-values (Benjamini–Hochberg correction, *padj*<0.05) and log2 fold-change thresholds (log2 fold change>0.58 unless otherwise indicated). Gene symbols were used for all downstream overlap analyses. For each dataset, the overlap between DEGs and cluster-specific gene sets was quantified by counting the number of DEGs mapping to each cluster. Results were summarized as the number of upregulated and downregulated genes per cluster and visualized using bar plots. To statistically assess enrichment, Fisher's Exact tests were performed for each cluster independently, using all genes detected in the cluster dataset as the background. Enrichment analyses were conducted separately for upregulated and downregulated gene sets, testing whether DEGs were over-represented in a given cluster relative to the background. *P*-values were adjusted for multiple testing using the Benjamini–Hochberg method, and clusters with adjusted *P*-values <0.05 were considered significantly enriched. For each cluster, enrichment metrics included the number of overlapping genes, total cluster size, the percentage of cluster genes represented by the DEG set, and the percentage of the DEG set represented by that cluster. All analyses were performed in R using the tidyverse, dplyr, tidyr and ggplot2 packages.

### Single-nucleus RNA-seq data visualization

Processed single-nucleus RNA-seq datasets from human placenta (Keenen et al., 2025) were analyzed using the associated publicly available interactive online visualization tool. Uniform Manifold Approximation and Projection (UMAP) and feature plots were generated to assess PLAC1 expression across annotated human trophoblast cell types.

### RT-qPCR analysis

Total RNA was extracted using the TRIzol reagent (Thermo Fisher Scientific). RNA was converted to cDNA using the High-Capacity cDNA Reverse Transcription kit (Applied Biosystems). Complementary DNAs were diluted 1:10 and subjected to RT-qPCR analysis using SYBR green master mix (Thermo Fisher Scientific). Primer sequences for RT-qPCR analysis are provided in Table S9. *Gapdh* (rat samples) and *POLR2A* (human samples) transcript levels were used for normalization and relative mRNA expression was calculated using the ΔΔCt method.

### shRNA manipulated cells

Control, rat and human PLAC1-targeted shRNAs were prepared (control shRNA: Addgene plasmid #1864; Table S10), cloned into the pLKO.1 vector, packaged into lentivirus, and lentiviral particles produced in Lenti-X 293T Cell Line (632180, Thermo Fisher Scientific), as previously described (Chakraborty et al., 2016). Lenti-X 293T cells were transiently transfected using Attractene (301005, QIAGEN) with the following plasmids: shRNA-containing transducing vector, packaging system plasmids (pMDLg/pRRE and pRSV-Rev), and a vesicular stomatitis virus G envelope plasmid (pMD2.G). Cells were maintained in DMEM supplemented with 10% FBS, 100 U/ml penicillin and 100 μg/ml streptomycin, for 24 h. After that, cells were cultured in Basal Human TSC Medium (Complete medium without CHIR9902, A830, SB431542, valproic acid and Y27632), rat TSC medium or BeWo cell medium. Supernatants were collected after 24 h, filter-sterilized, and stored frozen at −80°C until used. TSCs maintained in the stem state or BeWo cells were exposed to lentiviral particles, selected with puromycin dihydrochloride (2 μg/μl; A1113803, Sigma-Aldrich) for 2 days, and then maintained in a lower concentration of the antibiotic (1 μg/ml). The efficiency of lentiviral-mediated transduction and puromycin selection was >95%. Puromycin was removed during trophoblast cell differentiation.

### Human CG enzyme-linked immunosorbent assay

Conditioned medium was collected after 6 days of STB-3D culture. Human CG was quantified using an enzyme-linked immunosorbent assay (HC251F, Calbiotech), according to the manufacturer's instructions. Measurements were normalized to total cell protein, which was determined using the Bio-Rad detergent compatible protein assay.

### Immunocytochemical analyses

Human TSCs cultured in STB-2D, EVT and TO-EVT conditions were fixed in 4% PFA for 15 min at room temperature, and washed with PBS (pH 7.4). Cells were then permeabilized in PBS containing 0.1% Triton X-100 and 0.3% BSA for 20 min. After blocking with 0.3% BSA solution in PBS for 30 min, cells were incubated overnight at 4°C with primary antibodies (diluted in blocking solution): rabbit polyclonal antibodies to human FURIN (ab3467, Abcam), phycoerythrin (PE)-conjugated anti-HLA-G antibody (1:300; ab24384, Abcam), mouse or rabbit monoclonal antibodies to CDH1 (1:250; 14472 and 3195S, Cell Signaling Technology), rabbit monoclonal antibody to CGB (1:500; ab324382, Abcam) and mouse monoclonal antibody to SCD1 (1:500; ab34164, Abcam). Primary antibodies were detected with Alexa Fluor 568 goat anti-rabbit IgG, (1:800; A10042, Thermo Fisher Scientific) or Alexa Fluor 488 goat anti-mouse IgG (1:800; A32723, Thermo Fisher Scientific) secondary antibodies, diluted in blocking solution and incubated for 1 h at room temperature. Nuclei were visualized using DAPI. Images were captured with 2037a inverted Eclipse TiE with A1R confocal or 2047 Zeiss Axio Observer 7 with Apotome III and AI Sample Finder and processed using NIH ImageJ software.

### Cell fusion

A split green fluorescence protein (GFP) system (GFP11-labeled histone H2B and GFP1-10) was employed to assess cell fusion efficiency (Shao et al., 2014). Lentiviral particles expressing GFP11-H2B or GFP1-10 were produced and transduced into human TSCs creating two separate cell pools. These pools were then combined (total 200,000 cell/well) and cultured in STB-2D conditions for 5 days. Differentiated cells were fixed with 4% PFA for 15 min, counterstained with DAPI, and images captured with a 2047 Zeiss Axio Observer 7 with Apotome III and AI Sample Finder. The GFP and DAPI stained nuclei were quantified using NIH ImageJ software, and cell fusion efficiency was calculated as the percentage of GFP-positive nuclei over the total DAPI-positive nuclei.

### BrdU incorporation assay

Cell proliferation was assessed using a BrdU incorporation assay with the EZ-BrdU™ Cell Proliferation Assay Kit (Tonbo Biosciences), following the manufacturer's instructions with minor adaptations for a 24-well plate format. Briefly, cells were cultured in 24-well plates under the indicated experimental conditions until the confluence was reached. BrdU was added directly to the culture medium at the concentration recommended by the manufacturer and incubated for 6 h in stem state or 48 h in STB-2D conditions at 37°C in a humidified 5% CO₂ atmosphere. Following BrdU incorporation, cells were fixed and permeabilized according to the kit protocol. DNA was subsequently denatured to allow antibody access, and incorporated BrdU was detected by immunocytochemistry using the anti-BrdU antibody provided in the kit. Nuclei were counterstained with DAPI. After washes with PBS, images were captured using Zeiss Axio Observer 7 with Apotome III and AI Sample Finder. BrdU-positive and total DAPI-positive nuclei were quantified using NIH ImageJ. The percentage of BrdU-positive cells was calculated by normalizing BrdU-positive nuclei to the total number of nuclei per field. At least three independent fields per condition were analyzed for each experiment.

### Molecular modeling

Protein–protein interaction models were generated using AlphaFold 3 (Abramson et al., 2024) and assessed based on the weighted sum of pTM and piTM values (the AlphaFold-Multimer ranking metric), pLDDT scores and visual inspection. Model PDB files were visualized using PyMOL (PyMOL Molecular Graphics System, version 3.1, Schrödinger, LLC). The full-length, activated form of FURIN was used for the modeling.

### FURIN enzymatic activity

FURIN protease activity was determined using a FURIN Protease Assay Kit (78040, BPS Bioscience), according to the manufacturer's instructions. The assay measures FURIN-dependent cleavage of a flurogenic substrate (tert-butyloxycarbonyl-Arg-Val-Arg-Arg-7-amino-4-methylcoumarin). Cell lysates were prepared in assay buffer containing 1% Triton X-100. Protein concentrations were determined using the DC Protein Assay (5000112, Bio-Rad). Chloromethylketone, a FURIN protease inhibitor, was utilized as a control (provided by kit; 78040, BPS Bioscience).

### Treatment of human TSCs with a FURIN inhibitor

Human TSCs were treated with FURIN inhibitor, Decanoyl-RVKR-CMK (25 and 50 μM; 3501/1, R&D Systems) during 6 days of STB-2D differentiation (see above).

### Statistical analysis

Data analyses were performed using Prism GraphPad software. Results were determined as statistically significant when *P<*0.05*.* Details of statistical analyses used for each experiment are presented in the respective figure legends.

## Supplementary Material

10.1242/develop.205290_sup1 Supplementary information

Table S1. Differentially regulated transcripts identified in the RNA-seq analysis of rat placenta at gestation day 13.5 from wild type and PLAC1 mutant rats.

Table S2. Differentially regulated transcripts identified in the RNA-seq analysis of junctional zone tissue at gestation day 18.5 from wild type and PLAC1 mutant rats.

Table S3. Differentially regulated transcripts identified by RNA-seq analysis in rat TS cells following Plac1 knockdown and 15 days of differentiation.

Table S4. Differentially regulated transcripts identified by RNA-seq analysis in syncytiotrophoblast differentiated CT27 human TS cells following *PLAC1* knockdown.

Table S5. Differentially regulated transcripts identified by RNA-seq analysis in syncytiotrophoblast differentiated CT29 human TS cells following *PLAC1* knockdown.

Table S6. Differentially regulated transcripts identified by RNA-seq analysis in Choriocarcinomaderived BeWo cells following *PLAC1* knockdown.
