# Supplementary material for: Fundamental and unique roles of PLAC1 in the regulation of rat and human trophoblast cell development
Source: Development. 2026 May 12;153(9):dev205290. doi: 10.1242/dev.205290 (PMC13245914; doi:10.1242/dev.205290)
Supplement: Supplementary information [file develop-153-205290-s1.pdf]

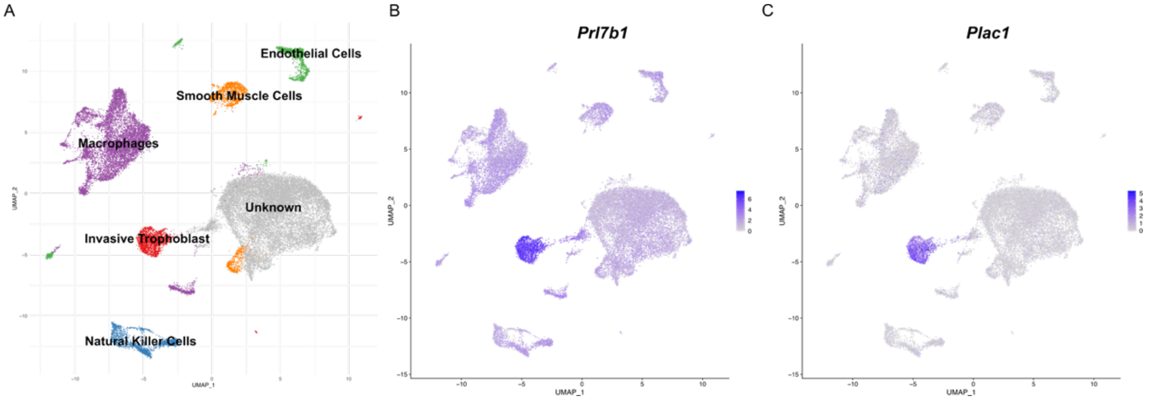

Fig. S1. Single cell RNA-sequencing of the gestation day 19.5 rat uterine-placental interface. **A)** Uniform Manifold Approximation and Projection (**UMAP**) visualization of cell clusters. **B** and **C**) UMAP plots show expression of *Prl7b1* and *Plac1*. Please note that *Prl7b1* and *Plac1* specifically localized to the invasive trophoblast cell cluster. The UMAP plots were generated from previously published datasets (Scott et al., 2022).

Development • Supplementary information

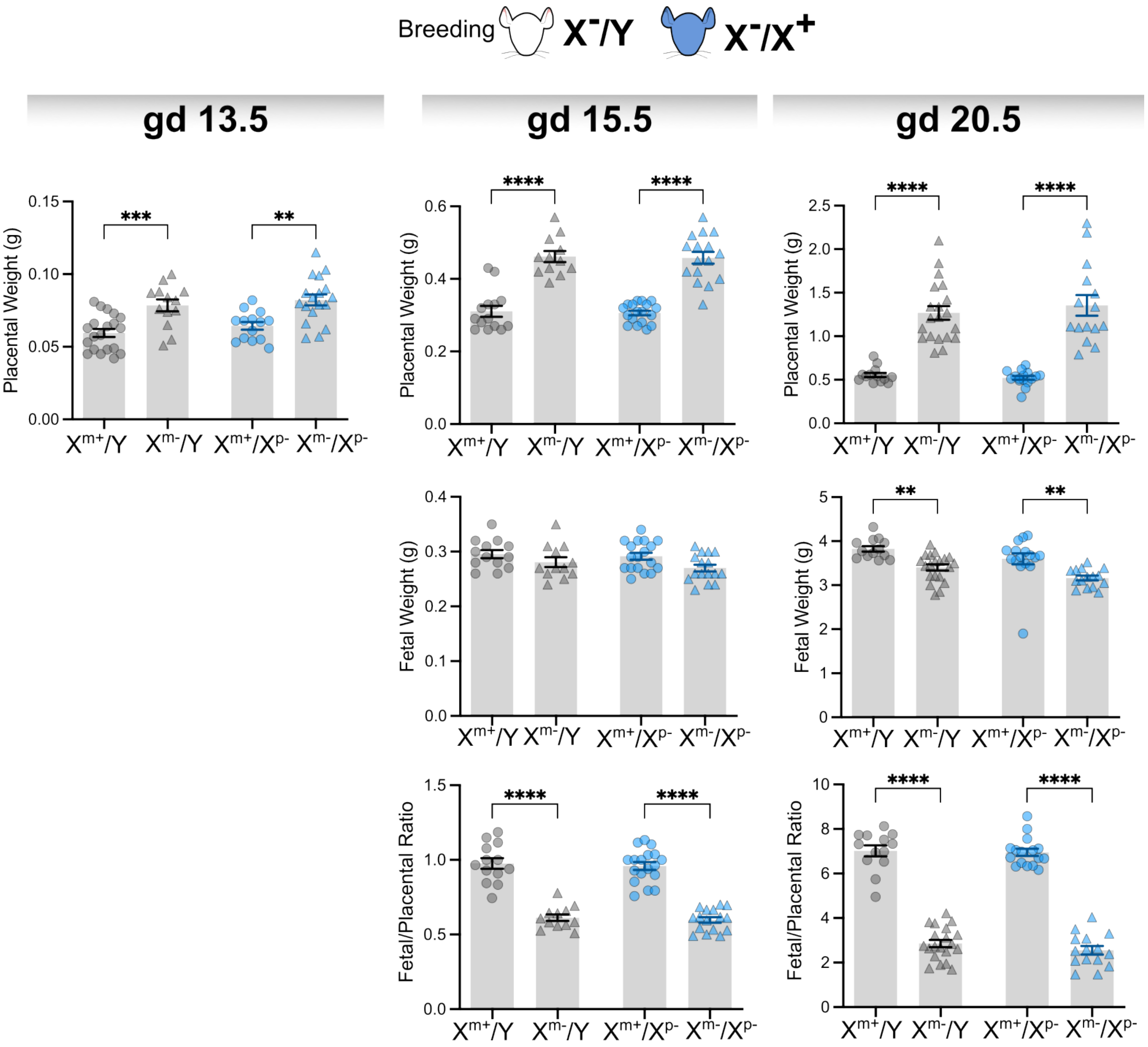

Fig. S2. The role of PLAC1 on placental and fetal size. Schematic representation of the breeding strategy and expected genotypes and phenotypes (**top**). Effects of *Plac1* disruption (X^{m-}) on placental weights on gestation day (**gd**) 13.5, 15.5 and 20.5 and fetal weights and placenta efficiency (fetus/placenta weight ratio) on gd 15.5 and 20.5. Data are presented as the mean ± SEM. Each dot represents a biological replicate. One-way analysis of variance and Holm-Sidak multiple comparison test: **$p<0.05$, ***$p<0.0005$, ****$p<0.0001$.

Development • Supplementary information

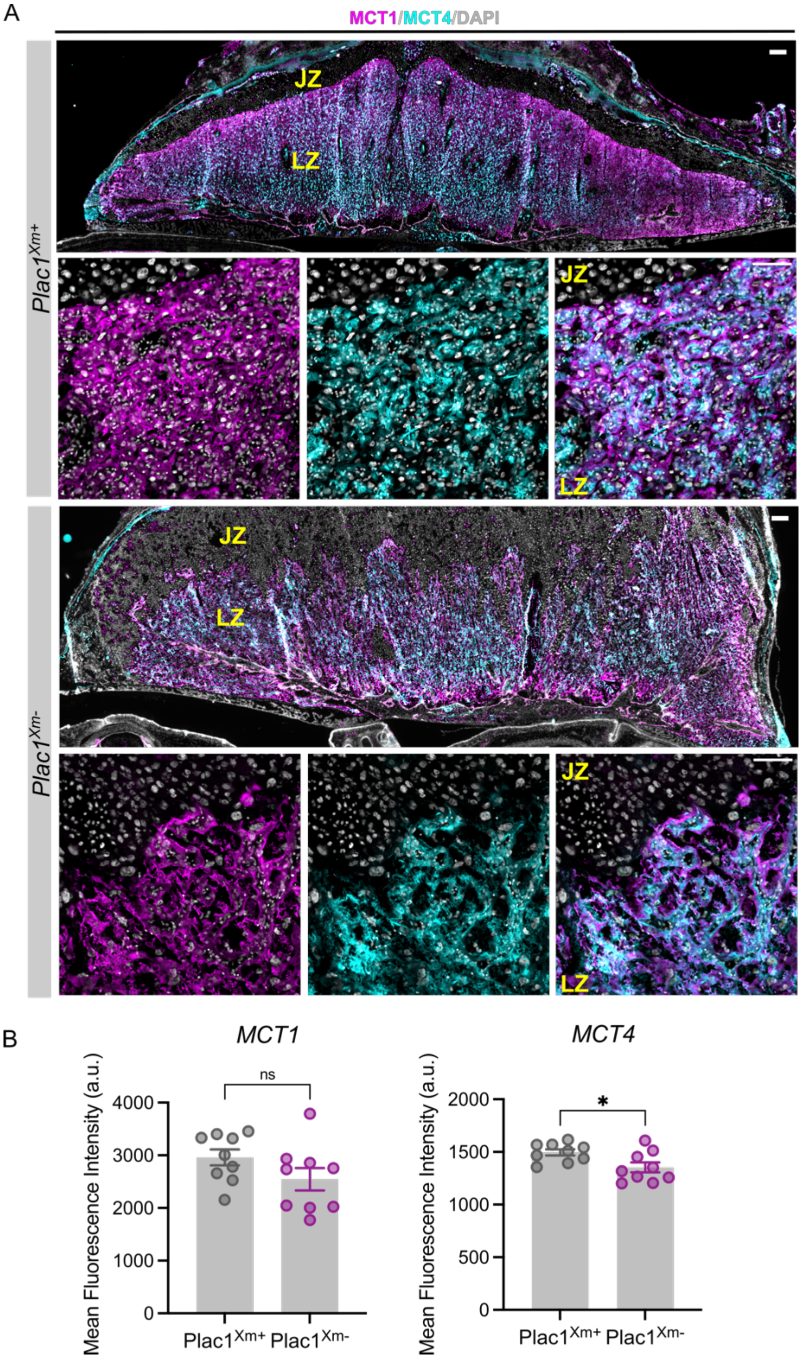

Fig. S3. Role of PLAC1 in labyrinth zone architecture. **A**) Representative immunostaining for monocarboxylate transporter 1 (**MCT1**) and monocarboxylate transporter 1 (**MCT4**) of wild type (*Plac1*$^{Xm+}$) and *Plac1* mutant (*Plac1*$^{Xm-}$) placentation sites at gestation day (**gd**) 18.5 (scale bar: 100 μm). **B**) Quantification of mean fluorescence for MCT1 and MCT4 of wild type (*Plac1*$^{Xm+}$) and *Plac1* mutant (*Plac1*$^{Xm-}$) placentation sites at gestation day (gd) 18.5. Measurements were performed in three randomly selected areas per sample across three independent experiments. Data are presented as the mean ± SEM. Each dot represents a biological replicate. Unpaired t-tests: *$p<0.05$. **Abbreviations**: **JZ**, junctional zone; **LZ**, labyrinth zone.

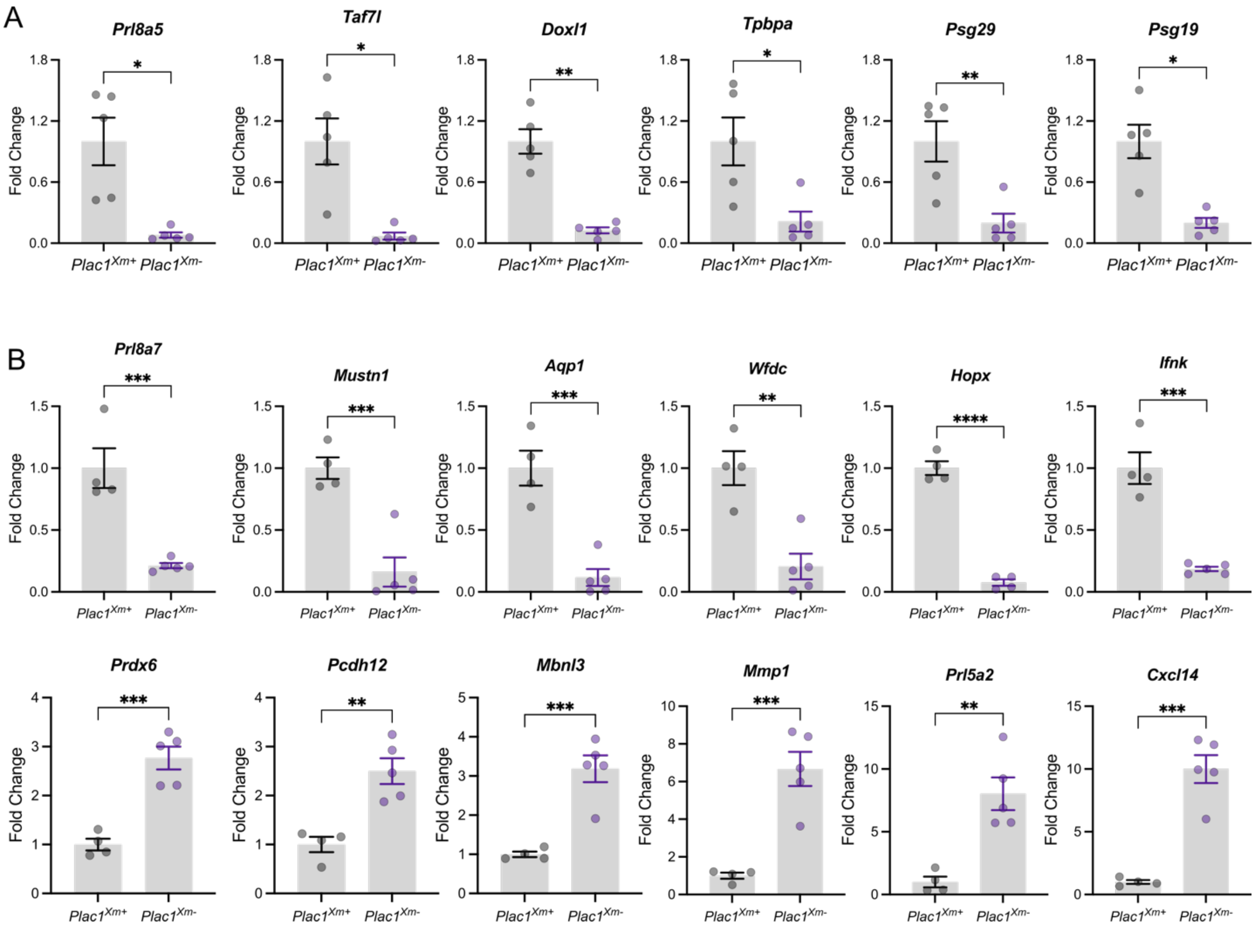

Fig. S4. RNA-sequencing (RNA-seq) analyses of wild type versus *Plac1* mutant placentation sites. A) RNA-seq validation of gestation day (**gd**) 13.5 placental specimens from wild type (*Plac1*$^{Xm+}$) and *Plac1* mutant placentas (*Plac1*$^{Xm-}$) using RT-qPCR. **B**) RNA-seq validation of gd 18.5 junctional zone specimens from wild type (*Plac1*$^{Xm+}$) and *Plac1* mutant placentas (*Plac1*$^{Xm-}$) using RT-qPCR. Data are presented as the mean ± SEM. Each dot represents a biological replicate. Unpaired t-tests: *p<0.05, **p<0.05, ***p<0.0005, ****p<0.0001.

Development • Supplementary information

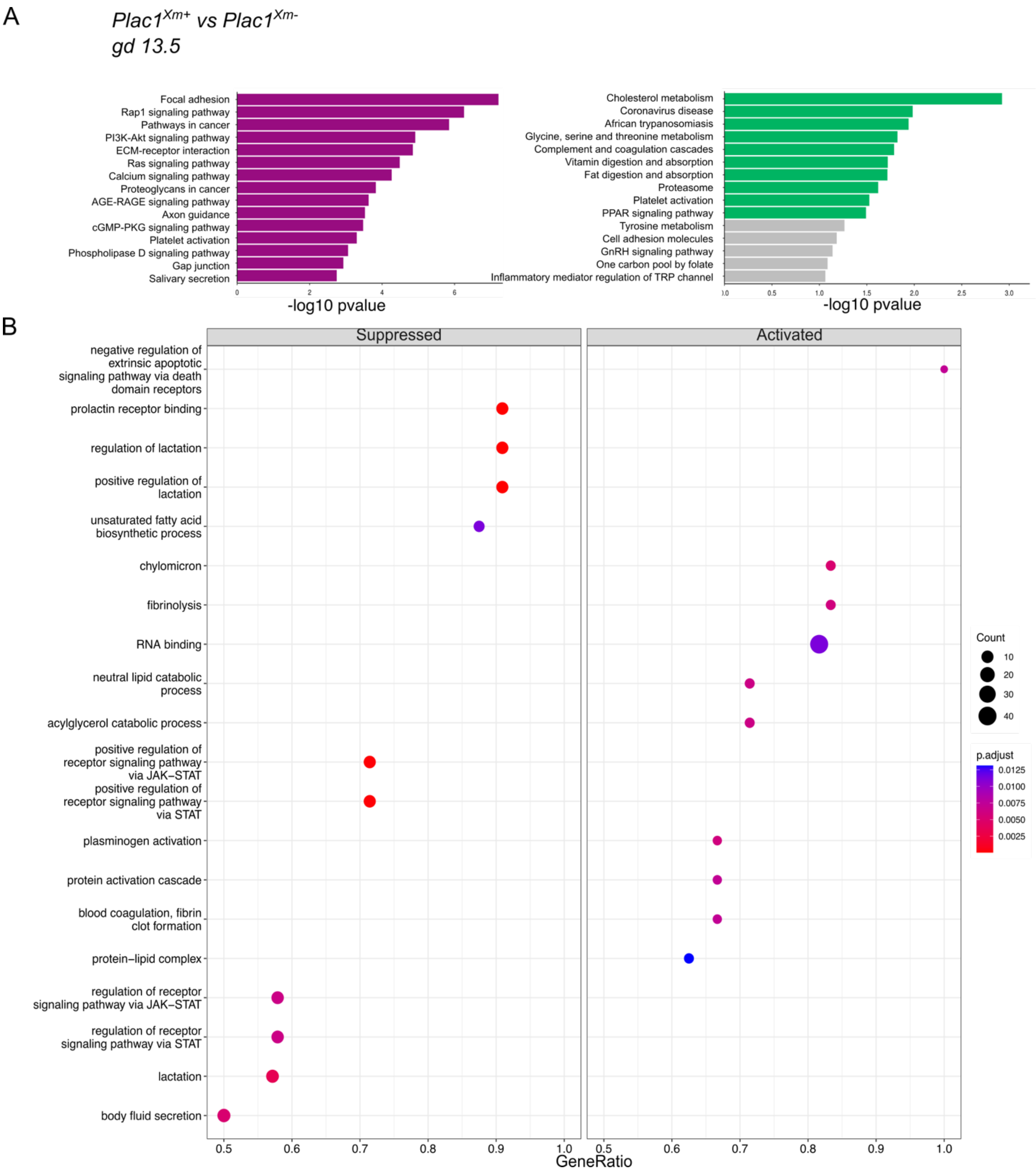

Fig. S5. Analysis of RNA-sequencing datasets from gestation day 13.5 wild type (*Plac1*$^{Xm+}$) and *Plac1* mutant (*Plac1*$^{Xm-}$) placental tissues. A) Kyoto Encyclopedia of Genes and Genomes enrichment analysis and **B**) Gene Set Enrichment Analysis.

Development • Supplementary information

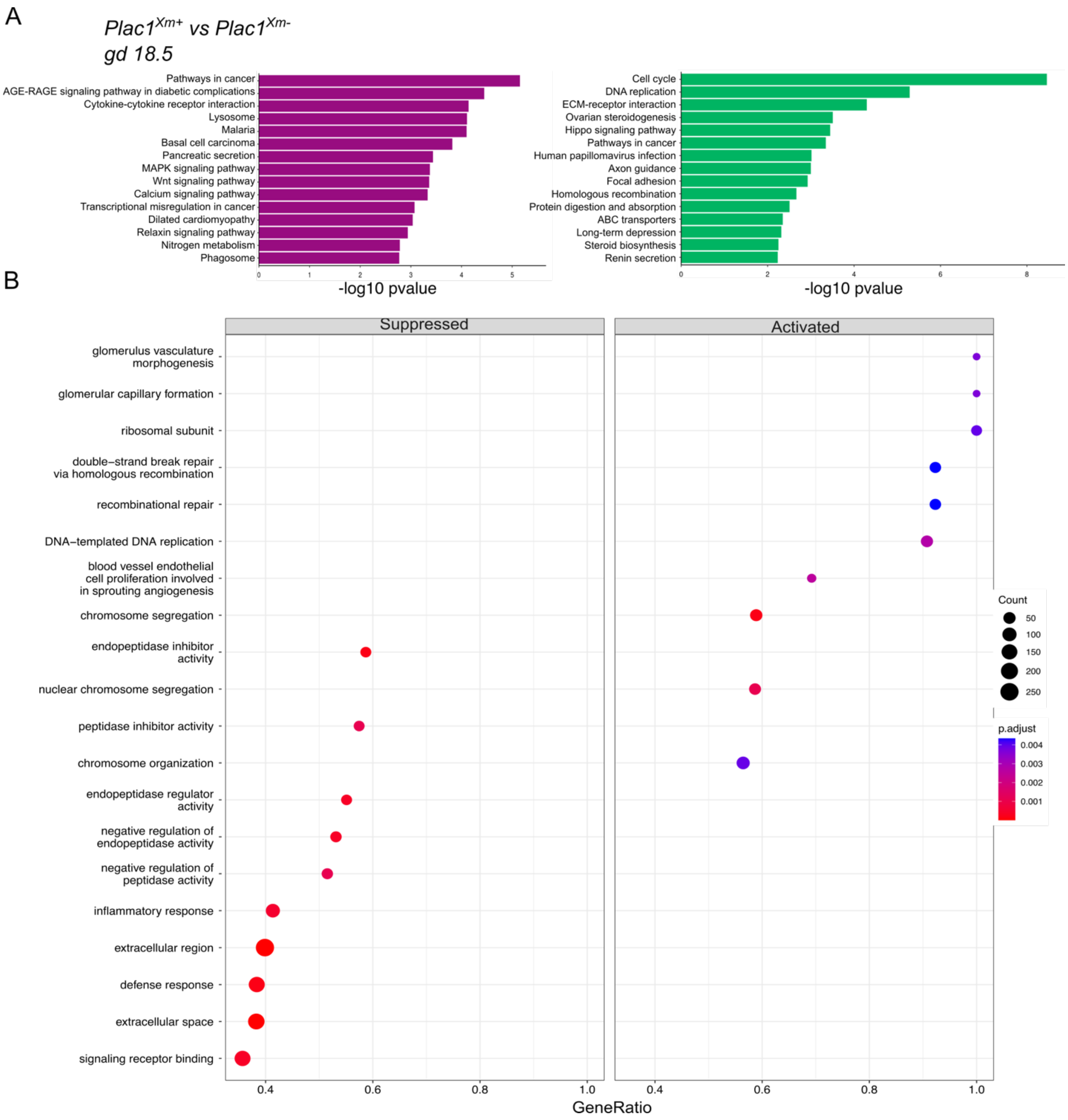

Fig. S6. Analysis of RNA-sequencing datasets from gestation day 18.5 wild type (*Plac1^{Xm+}*) and *Plac1* mutant (*Plac1^{Xm-}*) placental tissues. A) Kyoto Encyclopedia of Genes and Genomes enrichment analysis and **B)** Gene Set Enrichment Analysis.

Development • Supplementary information

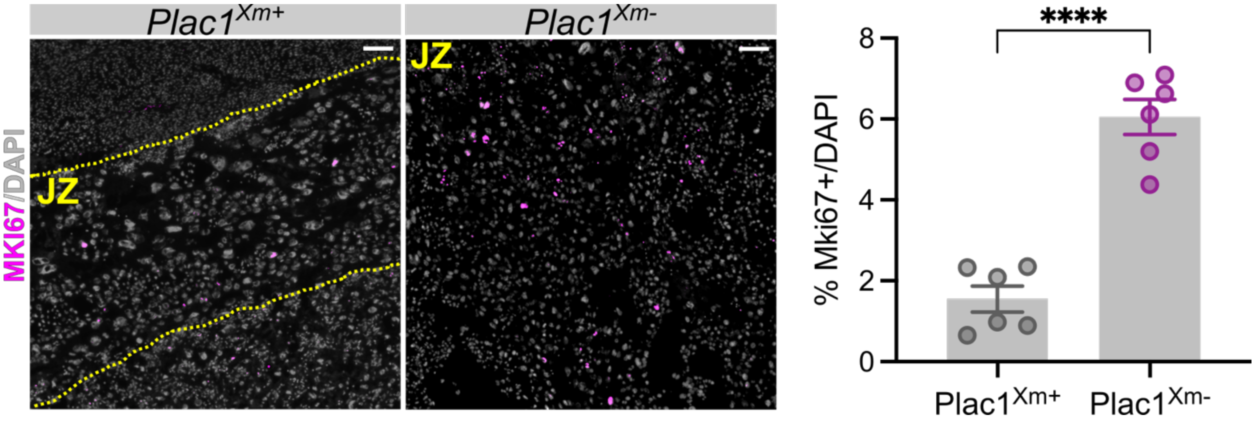

Fig. S7. Representative immunostaining for MKI67 and quantification expressed as percentage of Mki67 positive cells over total nuclei in the JZ of wild type (*Plac1*$^{Xm+}$) and PLAC1 mutant (*Plac1*$^{Xm-}$) placentation sites at gestation day (gd) 18.5 (scale bar: 100 µm). Abbreviation: **JZ**, junctional zone.

Development • Supplementary information

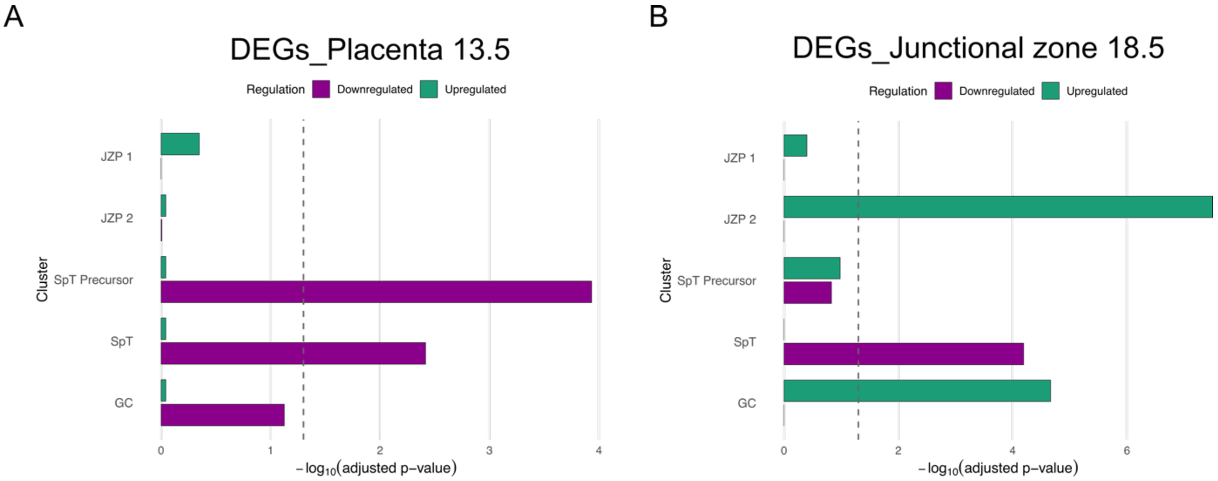

Fig. S8. Cluster enrichment analysis of PLAC1-responsive differentially expressed genes (DEGs). PLAC1-responsive DEGs identified from gestation day (**gd**) 13.5 placenta (**A**) and gd 18.5 junctional zone (**B**) were compared to trophoblast cell cluster–specific gene signatures established from single nucleus RNA-sequencing of the mouse placenta (Marsh and Blelloch, 2020). PLAC1-responsive DEGs were separated into upregulated and downregulated gene sets based on adjusted P value of < 0.05 and $\log_2$ fold-change thresholds. Enrichment of PLAC1-responsive DEGs within each cluster was assessed using Fisher's Exact test, with the set of all cluster-associated genes used as background. P values were corrected for multiple testing using the Benjamini–Hochberg method. Bar plots show $-\log_{10}$(adjusted P value) for each cluster, with dashed lines indicating the significance threshold (adjusted P=0.05). **Abbreviations**: **JZP1**, junctional zone precursor 1; **JZP2**, junctional zone precursor 2; **SpT Precursor**, spongiotrophoblast precursor; **SpT**, spongiotrophoblast; **GC**, glycogen cell.

Development • Supplementary information

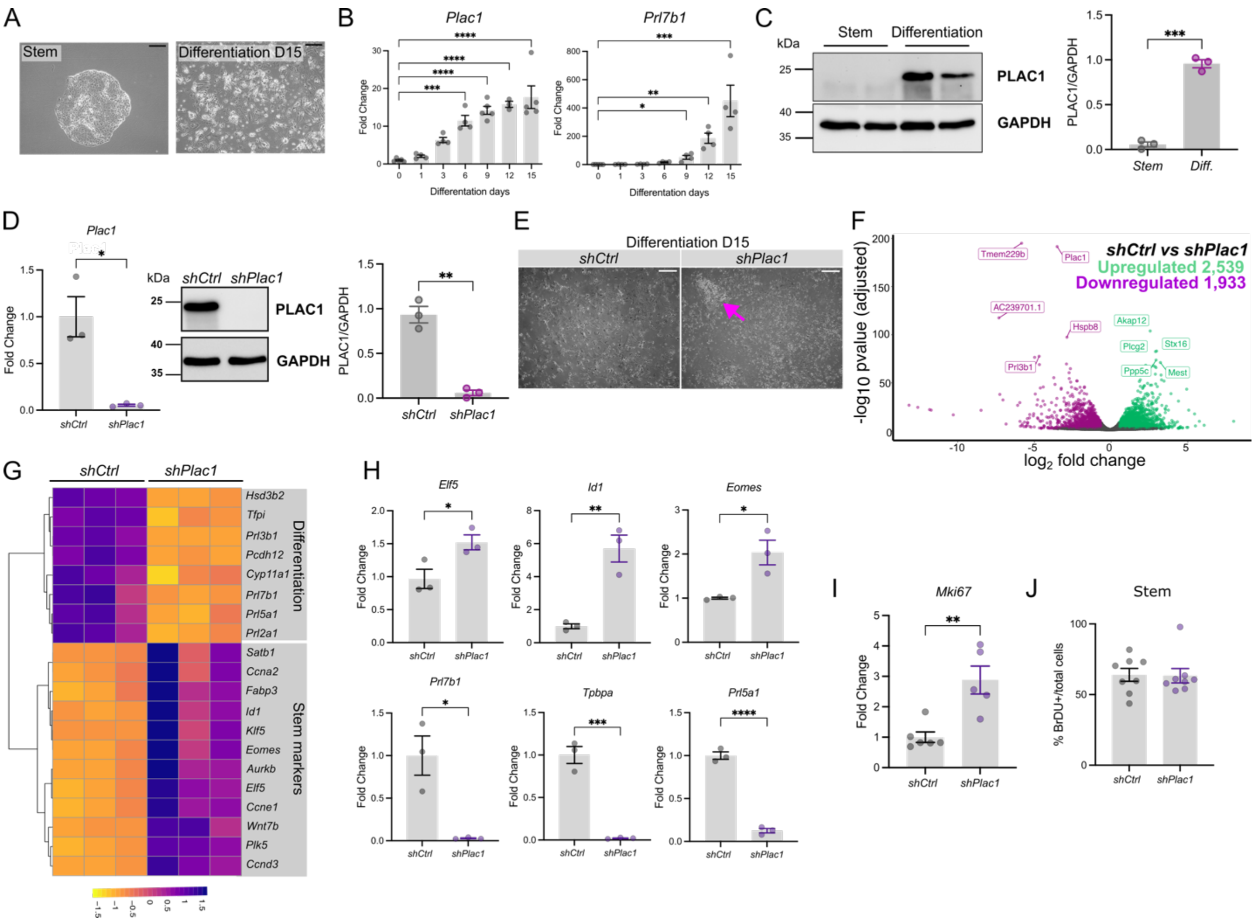

Fig. S9. Disruption of PLAC1 expression inhibited rat trophoblast stem (TS) cell differentiation. A) Representative phase contrast images of TS cell morphology in the stem state and after 15 days of differentiation (**D15**; scale bar: 200 µm). **B**) RT-qPCR measurement of *Plac1* and *Prl7b1* transcripts in rat TS cells throughout differentiation. **C**) Western blot analysis for PLAC1 in TS cells maintained in the stem state and following 15 days of differentiation. **D**) RT-qPCR and western blot analysis for PLAC1 in control shRNA (***shCtrl***) and *Plac1* shRNA (***shPlac1***) treated TS cells maintained in conditions promoting differentiation. **E**) Representative phase contrast images of control shRNA (***shCtrl***) and *Plac1* shRNA (***shPlac1***) treated TS cells maintained in conditions promoting differentiation (scale bar: 200 µm). The magenta arrow depicts a stem cell-like colony. **F**) Volcano plot of RNA-seq for *shCtrl* versus *shPlac1* treated TS cells maintained in conditions promoting differentiation. Most prominent differentially expressed genes are highlighted**. G**) Heatmap of selected of transcripts associated with the stem and differentiation states identified from RNA-seq of *shCtrl* versus *shPlac1* treated TS cells maintained in conditions promoting differentiation. **H**) RT-qPCR measurements of transcripts associated with the stem state and invasive trophoblast cell differentiation state identified from RNA-seq of *shCtrl* versus *shPlac1* treated TS cells maintained in conditions promoting differentiation. **I**) RT-qPCR measurements for *Mki67* transcripts from *shCtrl* versus *shPlac1* treated TS cells maintained in conditions promoting differentiation. **J**) BrdU incorporation in control shRNA (***shCtrl***) and *Plac1* shRNA (***shPlac1***) treated TS cells maintained in conditions promoting the stem state. Data are presented as the mean ± SEM. Each dot represents a biological replicate. Statistical analysis for **B**: one-way analysis of variance and Dunnett's multiple comparisons test; statistical analysis for **D**, **H**, **I**, and **J**: unpaired t-test, *p<0.05, **p<0.005, ***p<0.0005, ****p<0.0001.

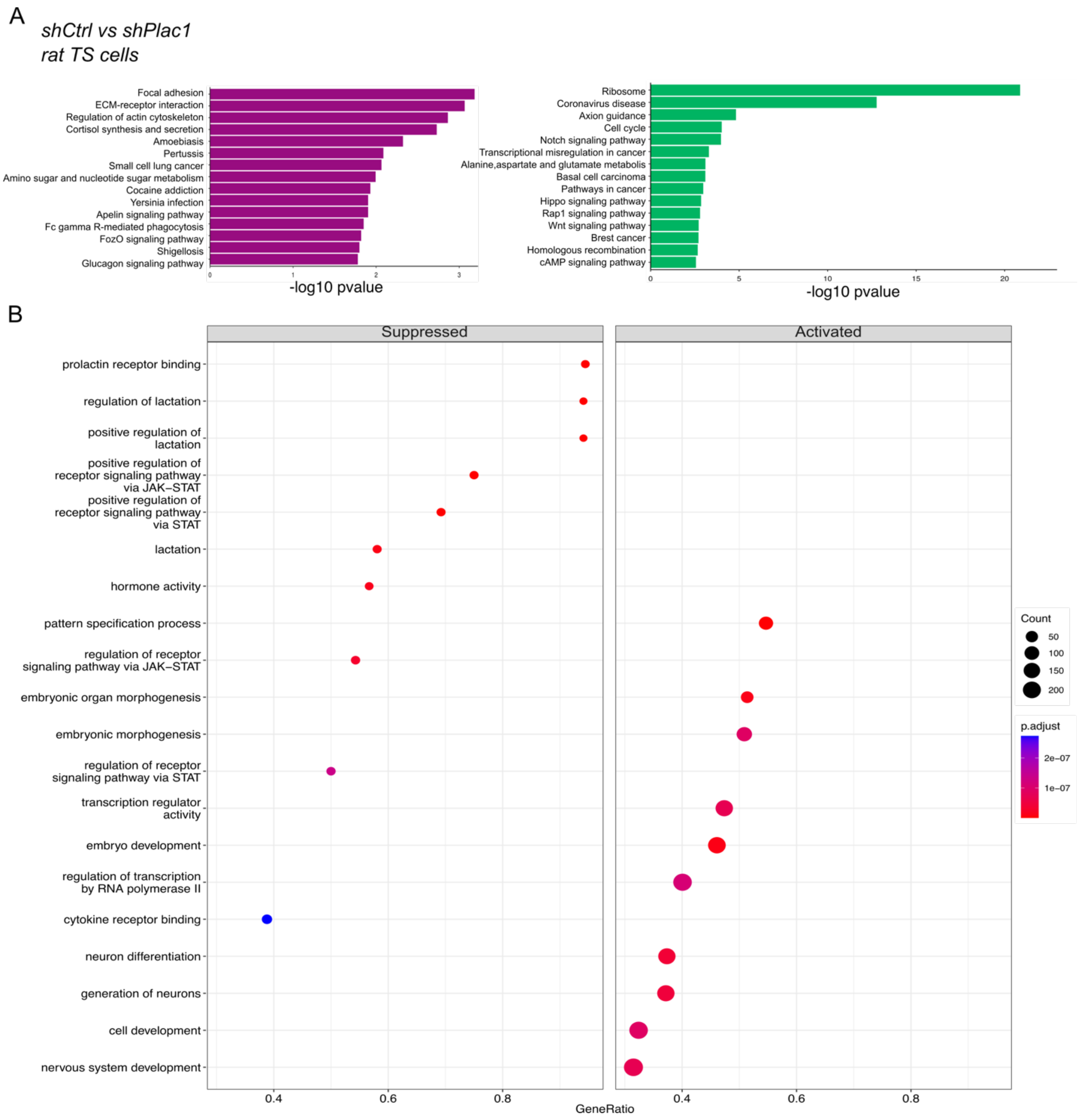

Fig. S10. Analysis of RNA-sequencing datasets from control shRNA (*shCtrl*) and *Plac1* shRNA (*shPlac1*) treated rat trophoblast stem cells maintained in conditions promoting differentiation. A) Kyoto Encyclopedia of Genes and Genomes enrichment analysis and **B)** Gene Set Enrichment Analysis.

Development • Supplementary information

A

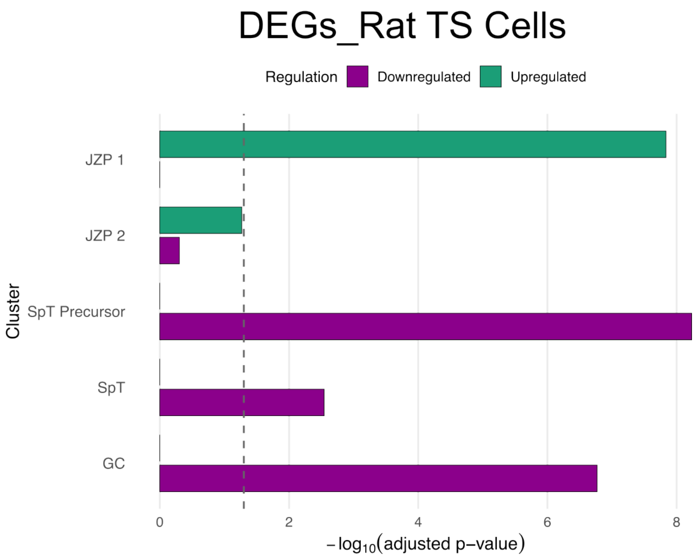

Fig. S11. Cluster enrichment analysis of PLAC1-responsive differentially expressed genes (DEGs) in rat trophoblast stem (TS) cells treated with control or *Plac1* shRNAs. PLAC1-responsive DEGs identified from control or *Plac1* shRNA treated TS cells were compared to trophoblast cell cluster–specific gene signatures established from single nucleus RNA-sequencing of the mouse placenta (Marsh and Blelloch, 2020). PLAC1-responsive DEGs were separated into upregulated and downregulated gene sets based on adjusted *P* value of < 0.05 and $\log_2$ fold-change thresholds. Enrichment of PLAC1-responsive DEGs within each cluster was assessed using Fisher's Exact test, with the set of all cluster-associated genes used as background. *P* values were corrected for multiple testing using the Benjamini–Hochberg method. Bar plots show $-\log_{10}$(adjusted *P* value) for each cluster, with dashed lines indicating the significance threshold (adjusted *P*=0.05). **Abbreviations**: **JZP1**, junctional zone precursor 1; **JZP2**, junctional zone precursor 2; **SpT Precursor**, spongiotrophoblast precursor; **SpT**, spongiotrophoblast; **GC**, glycogen cell.

Development • Supplementary information

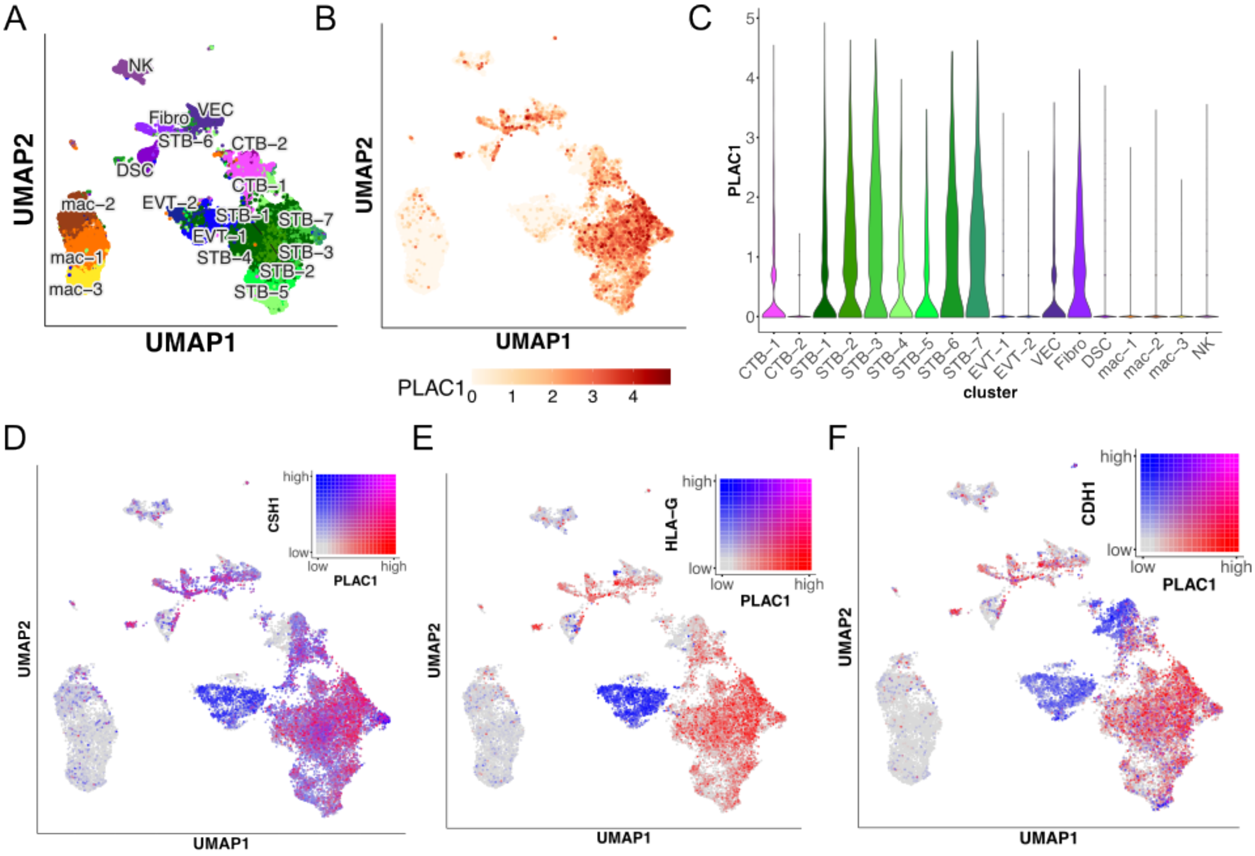

Fig. S12. PLAC1 expression in human trophoblast cell populations assessed by single-cell and single-nucleus RNA sequencing. A) Uniform Manifold Approximation and Projection (**UMAP**) representation of the integrated single-cell/snRNA-seq data from human term placenta tissue, with clusters annotated by specific trophoblast cell populations (Keenen et al., 2025). **B)** Feature plot showing PLAC1 expression. **C**) Violin plot showing PLAC1 expression in each cluster. **D**) Feature plot showing PLAC1 expression colocalized with the syncytiotrophoblast (**STB**) associated transcript, **CSH1**, indicating enrichment of PLAC1 in the STB population. **E**) Feature plot showing PLAC1 expression relative to the extravillous trophoblast (**EVT**) cell associated transcript, **HLA-G**, demonstrating absence of PLAC1 expression in EVT cells. **F)** Feature plot showing *PLAC1* expression relative to the cytotrophoblast (**CTB**) associated transcript, ***CDH1***, confirming lack of *PLAC1* expression in CTB. All plots were generated using the publicly available interactive online tool associated with the processed datasets (Keenen et al., 2025).

Development • Supplementary information

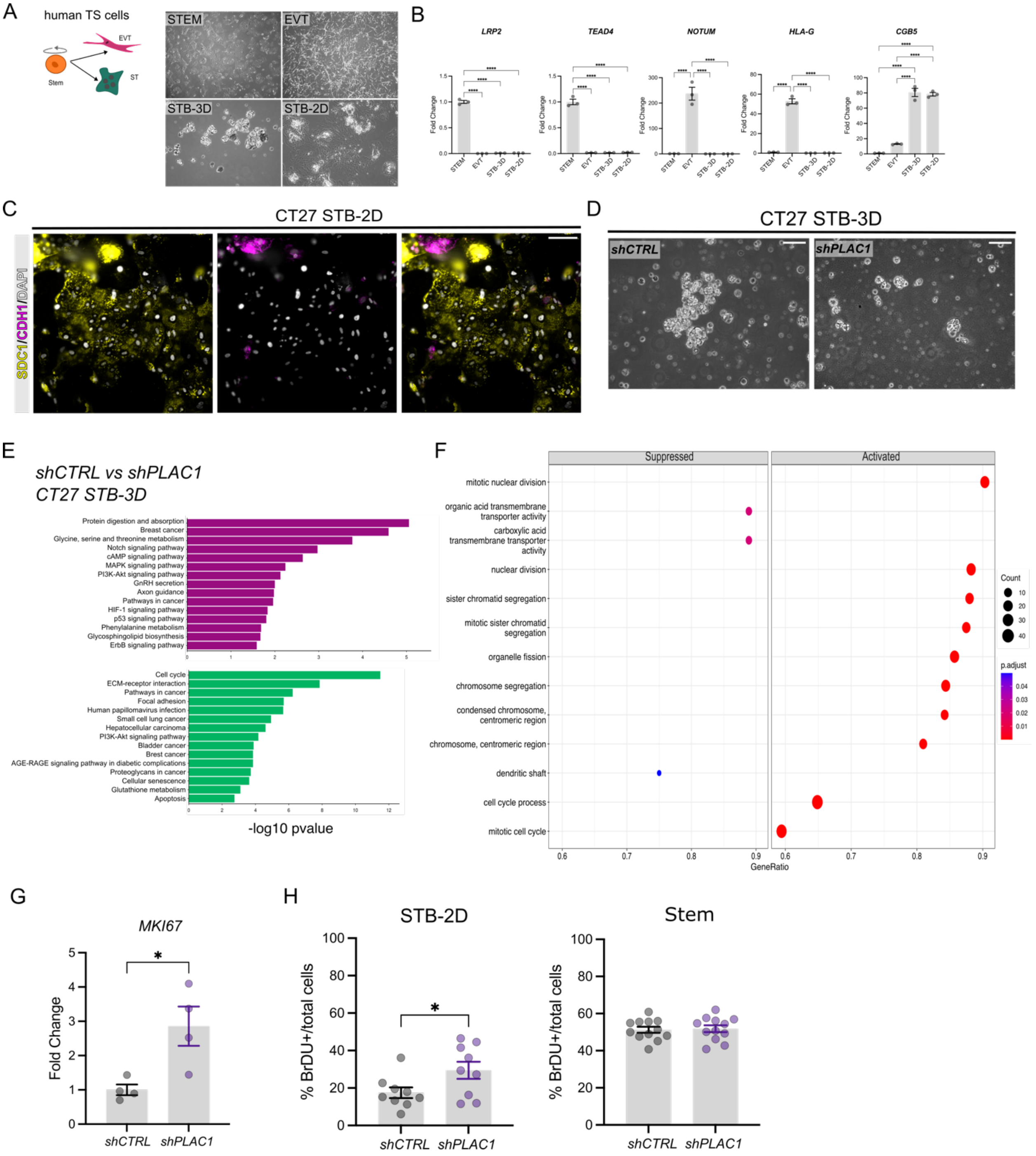

Fig. S13. Biology of PLAC1 in human trophoblast stem (TS) cells. A) Schematic diagram of human TS cells maintained in the stem state and following extravillous trophoblast (**EVT**) cell or syncytiotrophoblast (**STB**) differentiation. Representative phase contrast images of human TS cells in the stem state and following differentiation to EVT cells or STB. **B**) RT-qPCR measurements of stem cell and differentiated EVT and STB cell states of human TS cells. **C**) Representative images of SDC1 and CDH1 immunostaining in human CT27 TS cells in the STB differentiation state (scale bar: 100 µm). **D)** Representative phase contrast images of control shRNA (**shCTRL**) and *PLAC1* shRNA (***shPLAC1***) treated human CT27 TS cells in stem cell and STB differentiated states (scale bar: 200µm). **E**) Kyoto Encyclopedia of Genes and Genomes

enrichment analysis of RNA-seq datasets from *shCTRL* and *shPLAC1* treated human CT27 TS cells following exposure to conditions promoting STB differentiation. **F**) Gene Set Enrichment Analysis of RNA-seq datasets from *shCTRL* and *shPLAC1* treated human CT27 TS cells following exposure to conditions promoting STB differentiation. **G**) RT-qPCR measurement of *MKI67* in *shCTRL* and *shPLAC1* treated human CT27 TS cells in STB differentiated states. **H)** BrdU incorporation assay for *shCTRL* and *shPLAC1* treated human CT27 TS cells in STB-2D differentiated and stem cell states. Data are presented as the mean ± SEM. Each dot represents a biological replicate. Statistical analysis for B: One-way analysis of variance and Tukey's multiple comparisons test. **p<0.005, ****p<0.0001. Statistical analysis for G and H: unpaired *t*-test, *p<0.05.

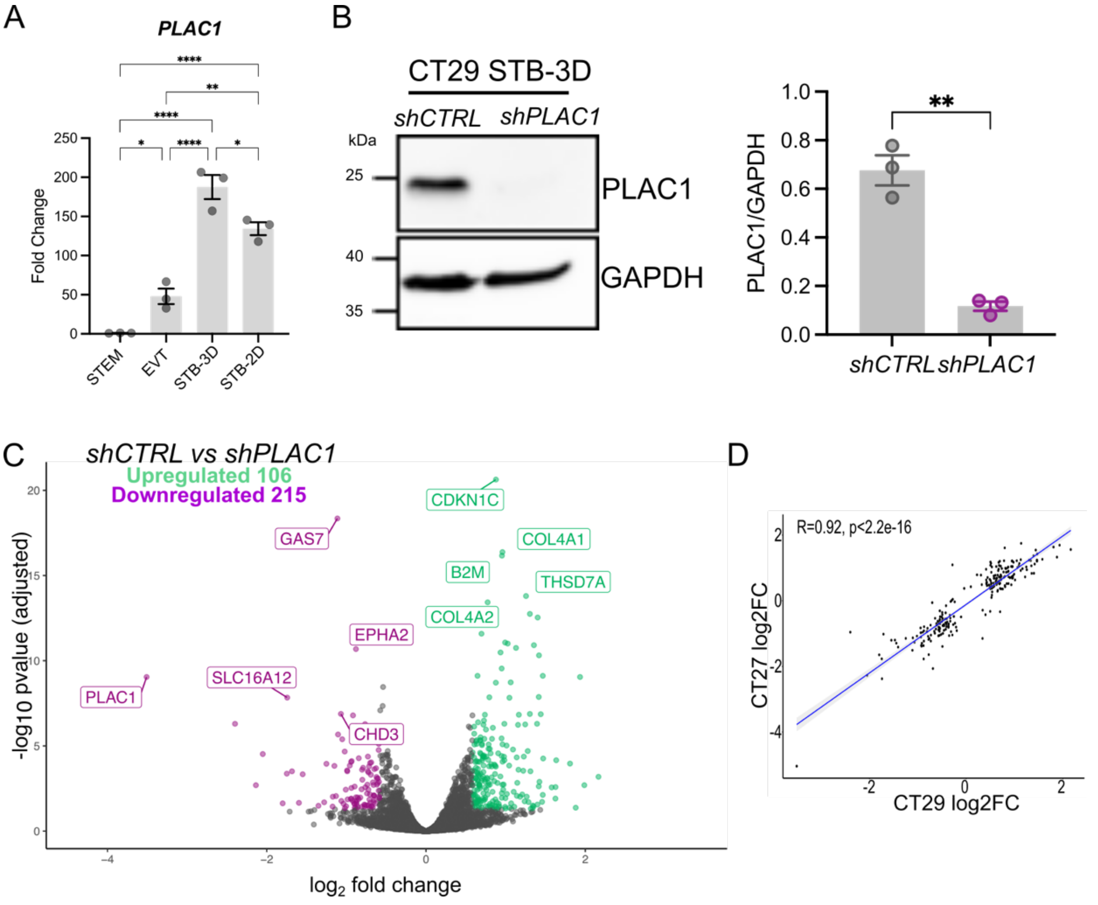

Fig. S14. Biology of PLAC1 in human CT29 trophoblast stem (TS) cells. **A**) RT-qPCR measurement of *PLAC1* expression in human CT29 TS cells maintained in stem and STB differentiated states. Data are presented as the mean ± SEM. Each dot represents a biological replicate. Statistical analysis for A: one-way analysis of variance and Tukey's multiple comparisons test. *p<0.05, **p<0.005, ****p<0.0001. **B**) Western blot analysis of PLAC1 expression in human CT29 TS cells in the STB-3D differentiated state treated with control shRNA (***shCTRL***) or PLAC1 shRNA (***shPLAC1***). **C**) Volcano plot of RNA-sequencing (**RNA-seq**) datasets from *shCTRL* and *shPLAC1* treated human CT29 TS cells in the STB-3D differentiation state. Most prominent differentially expressed genes are highlighted. **D**) Correlation of RNA-seq analyses for *shCTRL* and *shPLAC1* treated CT27 and *shCTRL* and *shPLAC1* treated CT29 human TS cells in the STB-3D differentiation state.

Development • Supplementary information

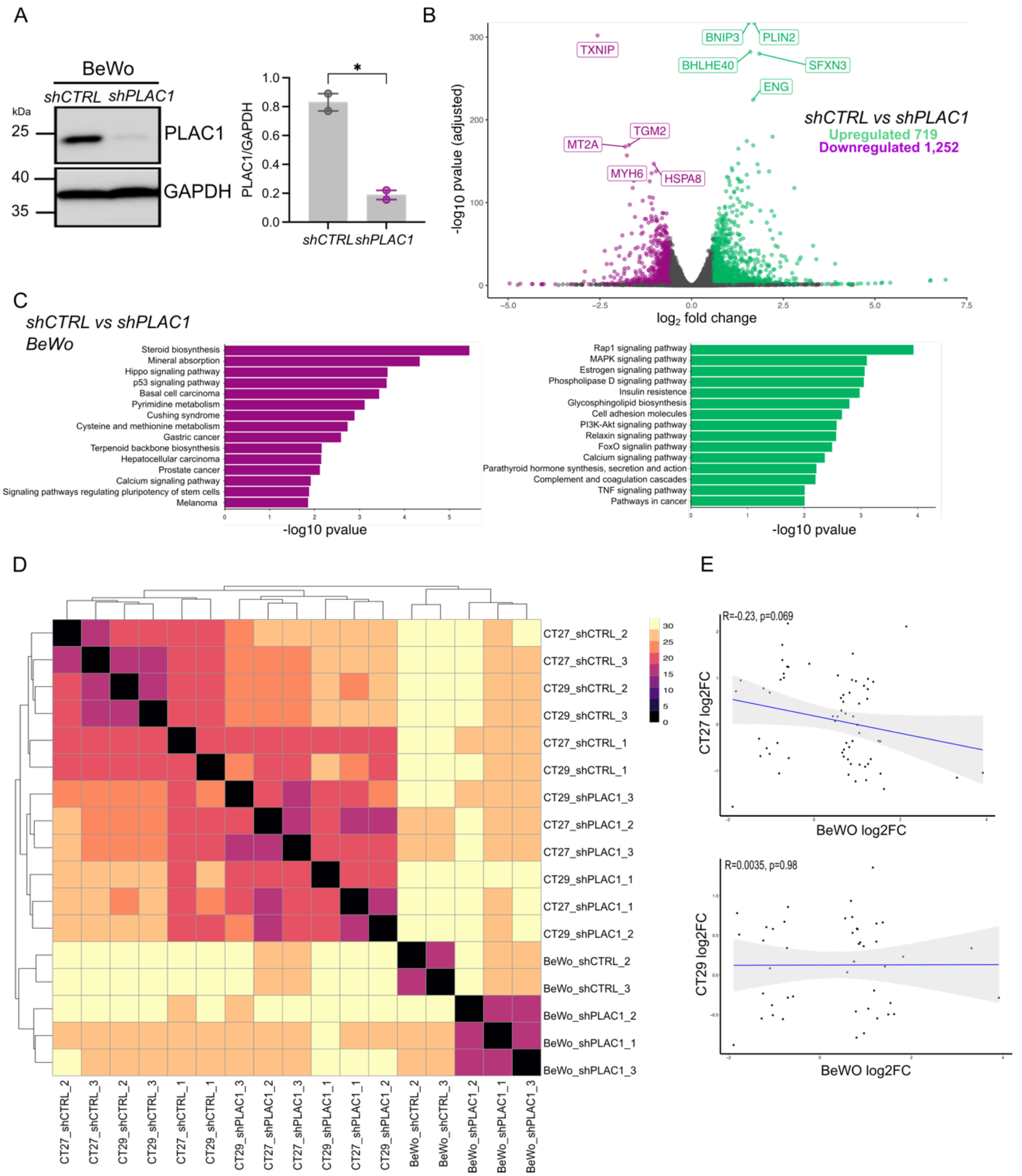

Fig. S15. Effects of disruption of PLAC1 in BeWo cells. A) Western blot analysis of control shRNA (***shCTRL***) and PLAC1 shRNA (***shPLAC1***) treated BeWo cells. **B**) Volcano plot of RNA-seq analysis of *shCTRL* and *shPLAC1* treated BeWo cells. Most prominent differentially expressed genes are highlighted. **C**) Kyoto Encyclopedia of Genes and Genomes enrichment analysis for RNA-sequencing (**RNA-seq**) datasets from *shCTRL* and *shPLAC1* treated BeWo cells. **D and E**) Correlation plots comparing RNA-seq datasets from *shCTRL* and *shPLAC1* treated BeWo cells and human TS cells (CT27 and CT29).

Development • Supplementary information

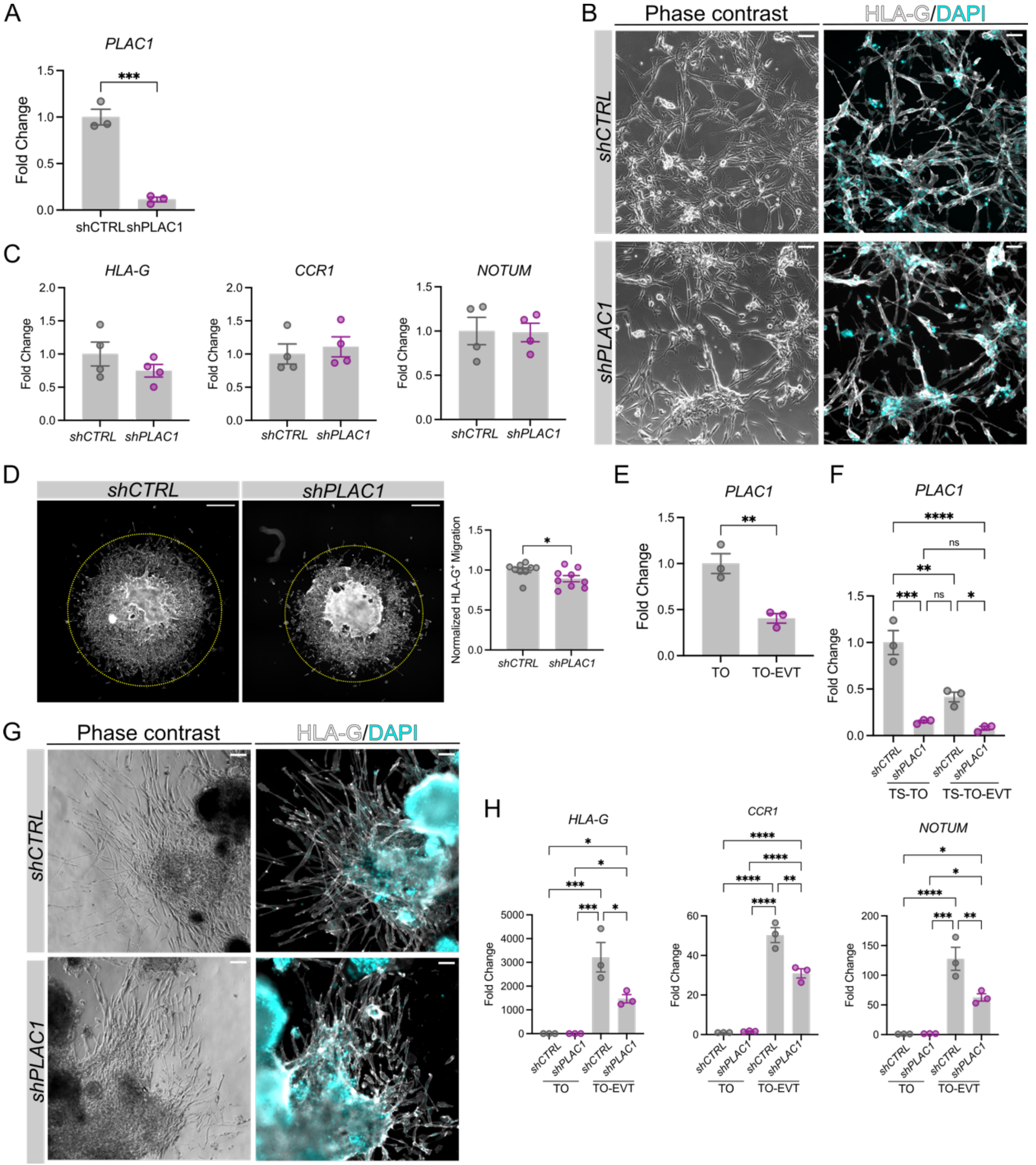

Fig. S16. Role of PLAC1 in EVT cell differentiation. A) RT-qPCR measurements of *PLAC1* transcript in *shCTRL* and *shPLAC1* treated human TS cells following exposure to conditions promoting EVT cell differentiation **B)** Representative images for phase contrast and HLA-G immunostaining of control shRNA (***shCTRL***) and *PLAC1* shRNA (***shPLAC1***) treated human CT27 TS cells in EVT cell differentiated states (scale bar: 100 µm). **C)** RT-qPCR measurements of EVT cell associated transcripts in *shCTRL* and *shPLAC1* treated human TS cells following exposure to conditions promoting EVT cell differentiation. **D)** Representative images and quantification of migration assay with HLA-G immunostaining of *shCTRL* and *shPLAC1* treated human TS cells (scale bar: 1000 µm). **E)** RT-qPCR measurements of *PLAC1* transcript in TS cell derived trophoblast organoids (**TOs**) and following exposure to conditions promoting EVT cell differentiation. **F)** RT-qPCR measurements of *PLAC1* transcripts in ***shCTRL*** and ***shPLAC1*** treated TOs and TOs after exposure to conditions promoting EVT cell differentiation (**TO-EVT**). **G)** Representative phase contrast and HLA-G immunostaining

images of control *shCTRL* and *shPLAC1* treated human TS cell derived trophoblast organoids (**TOs**) following exposure to conditions promoting EVT cell differentiation (scale bar: 100 μm). **H)** RT-qPCR measurements of EVT associated transcripts in ***shCTRL*** and ***shPLAC1*** treated TOs and TOs after exposure to conditions promoting EVT cell differentiation (**TO-EVT**). Data are presented as the mean ± SEM. Each dot represents a biological replicate. Statistical analysis for **B** and **C**: unpaired *t*-test, *p<0.05. Statistical analysis for **E:** One-way analysis of variance and Tukey's multiple comparisons test. *p<0.05,**p<0.005,***p<0.0005, ****p<0.0001.

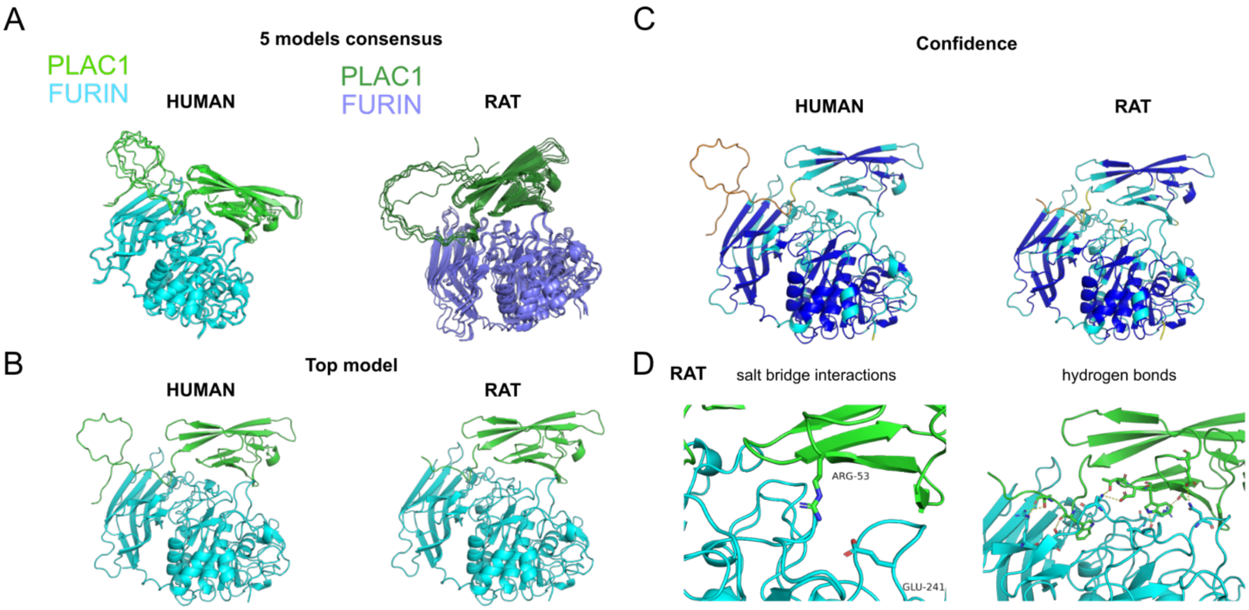

Fig. S17. Modeling PLAC1 and FURIN interactions. (**A-D**) Predicted PLAC1 and FURIN interaction determined by AlphaFold3. **A**) Five different models generated for human and rat PLAC1-FURIN interactions show a consensus in binding mode. **B**) Top PLAC1-FURIN interaction model generated from human and rat. **C**) Confidence for PLAC1-FURIN interaction models: Dark Blue > Cyan > Yellow > Orange. **D**) Rat PLAC1-FURIN salt bridge and hydrogen bond interactions.

Development • Supplementary information

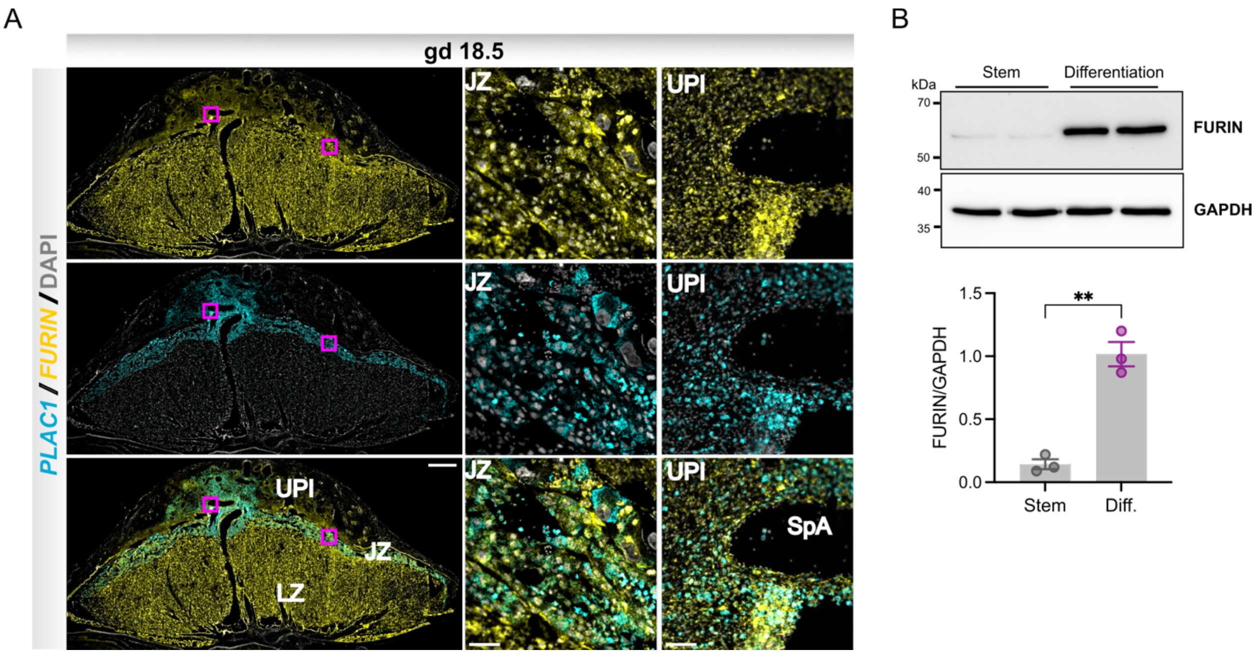

Fig. S18. PLAC1 and FURIN interaction in rat placenta and trophoblast stem (TS) cells.

A) Representative images of *Plac1* (cyan) and *Furin* (yellow) transcripts detected by *in situ* hybridization in rat placentation site at gestation day 18.5 (scale bar: 500 µm). Magenta outlined boxes in the left panels are shown at higher magnification in the respective right panels (scale bar: 100 µm). **B)** FURIN western blot for rat TS cells maintained in the stem state or induced to differentiate. GAPDH was used as a loading control.

Development • Supplementary information

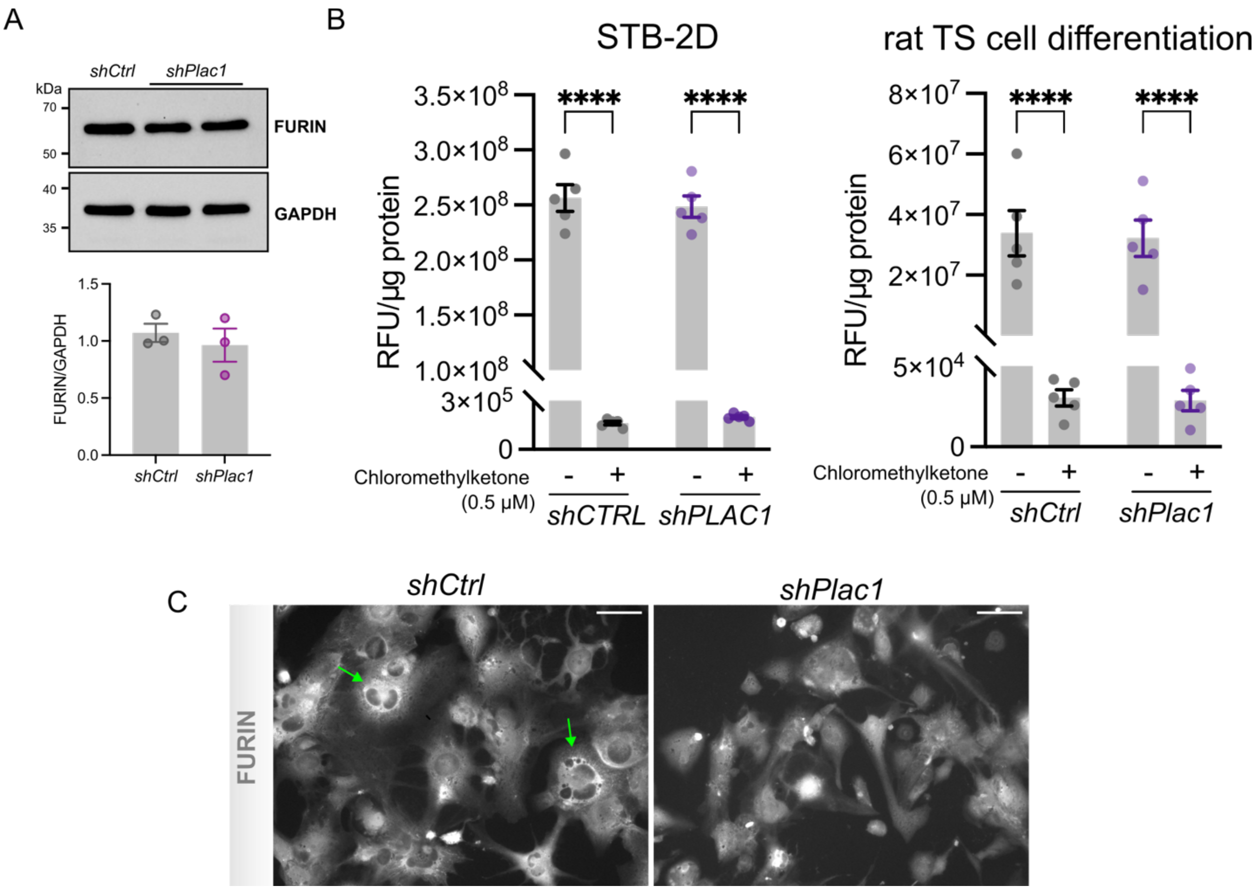

Fig. S19. PLAC1 and FURIN interactions in rat and human trophoblast stem (TS) cells. A) Western blot analyses of FURIN in control shRNA (***shCtrl***) and PLAC1 shRNA (***shPlac1***) treated rat TS cells following 15 days of differentiation. **B)** FURIN activity in *shCTRL* and *shPLAC1* treated human TS cells following STB-2D differentiation and FURIN activity in *shCTRL* and *shPLAC1* treated rat TS cells following 15 days of differentiation. Chloromethylketone (0.5 µM), a FURIN inhibitor, was used as a control to assess FURINspecific protease activity. Data is presented as the mean ± SEM. Each dot represents a biological replicate. Two-way analysis of variance and Tukey's multiple comparison test, ****p<0.0001. C) Representative images of FURIN detected by immunostaining in ***shCTRL*** and ***shPLAC1*** treated rat TS cells following 15 days of differentiation (scale bar: 100 µm). Green arrows depict FURIN perinuclear localization.

Development • Supplementary information

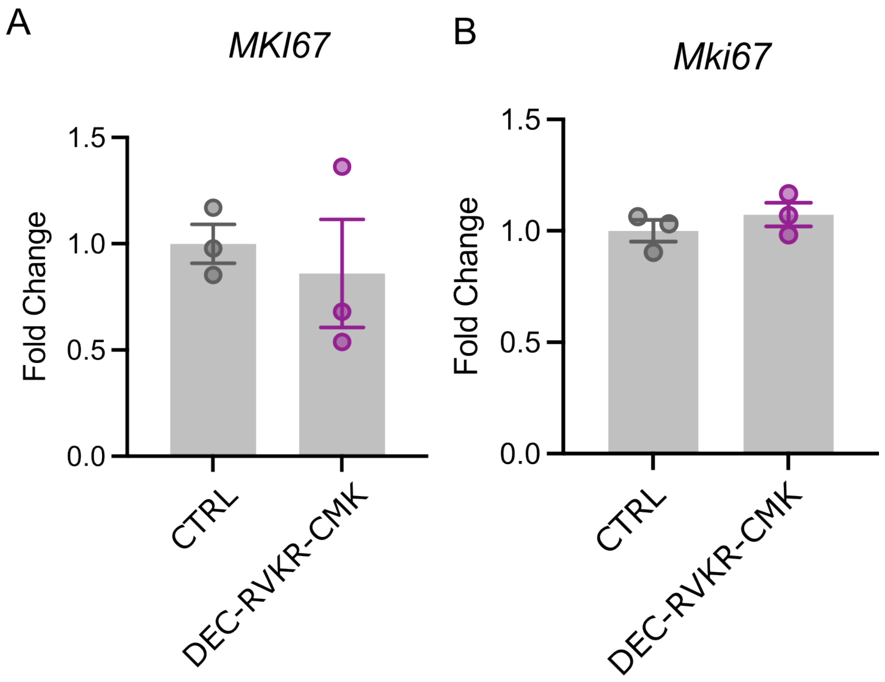

Fig. S20. Assessment of *MKI67* expression in human CT27 (A) and rat (B) trophoblast stem (TS) treated with a FURIN inhibitor (50 µM, DEC-RVKR-CMK) following exposure to conditions promoting differentiation. Data are presented as the mean ± SEM. Each dot represents a biological replicate. Data was analyzed with unpaired *t*-tests.

Development • Supplementary information

Table S1. Differentially regulated transcripts identified in the RNA-seq analysis of rat placenta at gestation day 13.5 from wild type and PLAC1 mutant rats.

Available for download at
https://journals.biologists.com/dev/article-lookup/doi/10.1242/dev.205290#supplementary-data

Table S2. Differentially regulated transcripts identified in the RNA-seq analysis of junctional zone tissue at gestation day 18.5 from wild type and PLAC1 mutant rats.

Available for download at
https://journals.biologists.com/dev/article-lookup/doi/10.1242/dev.205290#supplementary-data

Table S3. Differentially regulated transcripts identified by RNA-seq analysis in rat TS cells following *Plac1* knockdown and 15 days of differentiation.

Available for download at
https://journals.biologists.com/dev/article-lookup/doi/10.1242/dev.205290#supplementary-data

Table S4. Differentially regulated transcripts identified by RNA-seq analysis in syncytiotrophoblast differentiated CT27 human TS cells following *PLAC1* knockdown.

Available for download at
https://journals.biologists.com/dev/article-lookup/doi/10.1242/dev.205290#supplementary-data

Table S5. Differentially regulated transcripts identified by RNA-seq analysis in syncytiotrophoblast differentiated CT29 human TS cells following *PLAC1* knockdown.

Available for download at
https://journals.biologists.com/dev/article-lookup/doi/10.1242/dev.205290#supplementary-data

Table S6. Differentially regulated transcripts identified by RNA-seq analysis in Choriocarcinoma-derived BeWo cells following *PLAC1* knockdown.

Available for download at
https://journals.biologists.com/dev/article-lookup/doi/10.1242/dev.205290#supplementary-data

Table S7. Guide RNA sequences.

Name	Sequence
Plac1 F	AGAGTAGCCAGAGAACATGAGGG
Plac1 R	GTAGGGCTTAGTGAGCCAAGGGG

Table S8. Genotyping primers

Primer name	Sequence
Plac1 FW	ACCTGGTGCGATGGCTTACA
Plac1 RV	GGGGTTTACATGCTCTTCTGA
Kdm5c FW1	TTTGTACGACTAGGCCCCAC
Kdm5d FW2	TTGGTGAGATGGCTGATTCC
Kdm5d RV1	CCGCTGCCAAATTCTTTGG

Development • Supplementary information

Table S9. Primer sequences used for RT-qPCR

**Primer name**	**Sequence**	
**Rat**	Forward primer	Reverse primer
Gapdh	GACATGCCGCCTGCAGAAAC	AGCCCAGGATGCCCTTTAGT
Plac1	CCGTCTCTCCAGATGTCGTT	GAGCCCTTGGAAGCATAGTG
Prl5a1	TCCACACCAGACATTCCAGA	TTTCCAGGAAGCCAACATTC
Prl7b1	CCGTCATACTGTCTCAGCACATC	AGCTGTTGAGACCATTGACAACAAA
Tpbpa	AATAGCAAAAGTGACCAGGAGGAG	AAGTGACTGTGCTTGGTTTTCATC
Mmp12	GCACATTTTGATGAGGCAGA	TTGATTTTGGATTATTGGAATGC
Tfpi	GCCCGAGGAAGACGATGATA	TCCGCCTTCATTGCACAG
Psg29	GGCAGGGGATTCTACTCACA	GACTTGGGAAGTGGTAGACAGG
Psg19	TCAGGGTGTAGCCCTTGG	CAGGAGCCAGCAGGTTAAAA
Taf7l	GATCTCCTTCGGAATGTGGA	GCTAGGCTGAGGCTTTCCTT
Prl8a5	CCAAGGTCTTCCCTACAGCA	CCCAAGCAGACACGGTAAAT
Ifnk	GGTGGAAATAAGAAGATGTTTCAGT	CACAACTTCTATCTAAGTTCTTGCTTG
Wfdc1	GGACAACAGAGGCACTTTCC	AGTGTGTGGAAAGCACAGGA
Aqp1	CACTTGGCCGAAATGACC	AGGGTGCCAATGATCTCAA
Mustn1	CAGATCTTTGCCTGTGGCTA	GATGGGGGCTTCAGGAGT
Cxcl14	GCGAGGAGAAGATGGTTATCA	GCTTAGGATGCAGGCAGTG
Prl5a2	CTGAGGTGCAAAATGATCCA	TCCCATCTGGTTCTGAGCAT
Mmp1	GGTGATATTGTGTTCGCCTTC	TCAGGTCCATCAAATGGGTTA
Mbnl3	CATGTTCGCCCAGCAGAT	TGGATTCATAGGAAAAGATGCAA
Pcdh12	AGAACGGAACCCTGTGGAG	GACAGCAGCTGGGAGATTTG
Prdx6	TTGATTGCTCTTTCAATAGACTCTG	CTGCACCATTGTAAGCATTGA
Prl8a7	GAGCTGCCATTGAGTCAACA	CAGCTTTATGGATGGCACTG
Mki67	AGTGGCCAAACAGACTTGCT	AGGCACTCCCTCACTCTTGT

Development • Supplementary information

Prl8a9	CCATTCGACTCTCTCAAACTCC	GATCCAGGCACCCACAAAA
Doxl1	CAACTGCCAGGCTACCAGAA	GACCACAGGGGGATTCCAAG
Eomes	GGCAAAGCGGACAATAACAT	GCTGGGTGATATCCGTGTTT
Hopx	ACCACGCTGTGCCTCATC	AGGCGCTGCTTAAACCATT
Id1	CTGAACGGCGAGATCAGTG	GGAGTCCATCTGGTTCCTCA
Elf5	CAAGACTGTCACAGCCGAACAA	TTCTTCCTTTGTCCCCACATC
Human		
PLAC1	GGACTGGCCCTCAAGACTTA	TGAAATGTGGCTTTGTGCTC
LRP2	CTGCTCCTGGCTCTCGTC	TCCCATCACACCTCCAGTCT
TEAD4	CAGGTGGTGGAGAAAGTTGAGA	GTGCTTGAGCTTGTGGATGAAG
NOTUM	ACAGGGATCCTGTCCTCACA	CTCCAAACATCACTGGAGCA
HLA-G	CCACCACCCTGTCTTTGACTAT	ACGTCCTGGGTCTGGTCCT
CGB5	AGCACTTTGCTCGGGTCA	GGCACAGGGAGTAGGGTGTA
SDC1	CTATTCCCACGTCTCCAGAACC	GGACTACAGCCTCTCCCTCCTT
POLR2A	TCCGTATTCGCATCATGAAC	TCATCCATCTTGTCCACCAC

Table S10. shRNA sequences used for loss-of-function experiments.

**Name**	**Sequence**
**Rat**	
shPlac1 F	ccggtTAGTCATCTGACTGTGCAAATctcgagATTTGCACAGTCAGATGACTAtttttg
shPlac1 R	aattcaaaaaTAGTCATCTGACTGTGCAAATctcgagATTTGCACAGTCAGATGACTAa
Human	
shPLAC1 F	ccggtGGTCTCCTGAAGATGCTATctcgagATAGCATCTTCAGGAGACCtttttg
shPLAC1 R	aattcaaaaaGGTCTCCTGAAGATGCTATctcgagATAGCATCTTCAGGAGACCa

Development • Supplementary information
